# Human intestinal organoids from Cronkhite-Canada syndrome patients reveal link between serotonin and proliferation

**DOI:** 10.1172/JCI166884

**Published:** 2023-11-01

**Authors:** Victoria Poplaski, Carolyn Bomidi, Amal Kambal, Hoa Nguyen-Phuc, Sara C. Di Rienzi, Heather A. Danhof, Xi-Lei Zeng, Linda A. Feagins, Nan Deng, Eduardo Vilar, Florencia McAllister, Cristian Coarfa, Soyoun Min, Hyun Jung Kim, Richa Shukla, Robert Britton, Mary K. Estes, Sarah E. Blutt

**Affiliations:** 1 Program in Translational Biology and Molecular Medicine,; 2 Department of Molecular Virology and Microbiology, and; 3 Alkek Center for Metagenomics and Microbiome Research, Baylor College of Medicine, Houston, Texas, USA.; 4 Department of Internal Medicine, Center for Inflammatory Bowl Diseases, The University of Texas at Austin Dell Medical School, Austin, Texas, USA.; 5 Department of Clinical Cancer Prevention, The University of Texas MD Anderson Cancer Center, Houston Texas, USA.; 6 Dan L Duncan Comprehensive Cancer Center and; 7 Department of Molecular and Cellular Biology, Baylor College of Medicine, Houston, Texas, USA.; 8 Department of Inflammation and Immunity, Lerner Research Institute, Cleveland Clinic, Cleveland, Ohio, USA.; 9 Department of Medicine, Section of Gasteroenterology and Hepatology, Baylor College of Medicine, Houston, Texas, USA.; 10 Department of Medicine, Section of Infectious Diseases, Baylor College of Medicine, Houston Texas, USA.

**Keywords:** Gastroenterology, Human stem cells, Molecular biology

## Abstract

Cronkhite-Canada Syndrome (CCS) is a rare, noninherited polyposis syndrome affecting 1 in every million individuals. Despite over 50 years of CCS cases, the etiopathogenesis and optimal treatment for CCS remains unknown due to the rarity of the disease and lack of model systems. To better understand the etiology of CCS, we generated human intestinal organoids (HIOs) from intestinal stem cells isolated from 2 patients. We discovered that CCS HIOs are highly proliferative and have increased numbers of enteroendocrine cells producing serotonin (also known as 5-hydroxytryptamine or 5HT). These features were also confirmed in patient tissue biopsies. Recombinant 5HT increased proliferation of non-CCS donor HIOs and inhibition of 5HT production in the CCS HIOs resulted in decreased proliferation, suggesting a link between local epithelial 5HT production and control of epithelial stem cell proliferation. This link was confirmed in genetically engineered HIOs with an increased number of enteroendocrine cells. This work provides a new mechanism to explain the pathogenesis of CCS and illustrates the important contribution of HIO cultures to understanding disease etiology and in the identification of novel therapies. Our work demonstrates the principle of using organoids for personalized medicine and sheds light on how intestinal hormones can play a role in intestinal epithelial proliferation.

## Graphical abstract

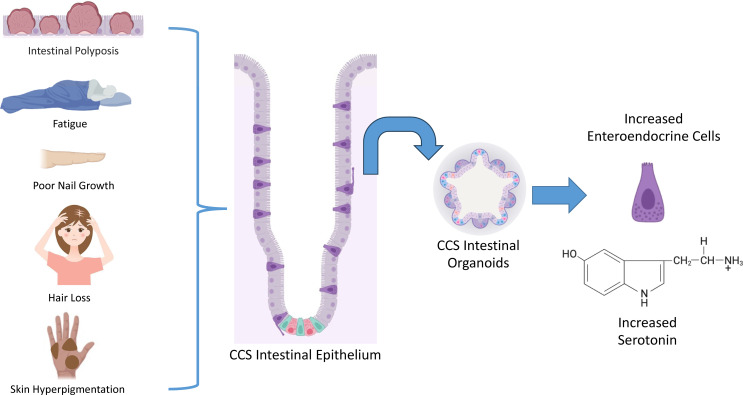

## Introduction

In 1955, Leonard Cronkhite and Wilma Canada documented a rare, nonhereditary disease associated with nonadenomatous cystic polyps in the gastrointestinal (GI) tract, ectodermal abnormalities of alopecia, nail dystrophy, and skin hyperpigmentation that was later termed Cronkhite-Canada Syndrome (CCS) (1–4). CCS presents with diarrhea, weight loss, abdominal pain, anorexia, hematochezia, nausea, vomiting, and dysgeusia (2, 5, 6). Since 1955, a little over 500 individuals worldwide have been diagnosed with CCS (7), classifying it as a rare disease using FDA criteria. Affecting individuals predominately in middle to late adulthood (2), the etiology has been difficult to elucidate due to the rareness of the disease. There is no link of germline mutations in CCS (8, 9); instead, immune dysregulation akin to autoimmune reactions triggered potentially by infection, vitamin deficiency, stress, and fatigue are thought to play a key role in the disease (8, 10, 11). Less than 5% of individuals enter remission from CCS-associated symptoms and the overall prognosis once diagnosed is poor. The pathology of the GI tract presents with edema of the lamina propria along with a marked mononuclear inflammatory cell infiltrate both in the polyp regions as well as in the intervening nonpolyp mucosa (12, 13). Interestingly, several groups report tortuous dilated gland/crypt regions that are architecturally distorted (12–14) suggesting dysregulation of the intestinal stem cell (ISC), which resides within the base of the gland/crypt (15), may be an important component of the etiology of the disease. The ultra-rare incidence of CCS has made the development and evaluation of therapeutic approaches difficult. Current approaches involve the use of drugs that suppress the immune system consistent with the autoimmune etiology. The development of human intestinal epithelial stem cell–derived organoids (HIOs), which function as ex vivo models of the GI tract (16, 17), now provides powerful tools with which to dissect the molecular mechanisms of human diseases. Resident ISCs from the small intestine or colon are isolated from biopsy samples or surgical specimens and cultured in the extracellular matrix matrigel. The cells proliferate and self assemble into 3-dimensional HIOs (18, 19). The presence of growth factors in the surrounding media promote cell division of the ISC, and, upon manipulation of these growth factors, the ISC differentiates into a heterogeneous population of mature cells that mimic functional intestinal epithelium (19–21). HIOs model many aspects of the physiology and regionality of the intestine, including stem cell regulation (22, 23), absorption (21, 24), and secretion (25). HIOs can be genetically manipulated (26–30) and are being used to investigate the role of individual genetic variability, including demographic factors in GI biology. Most importantly, HIOs generated from individuals that represent specific populations, including rare diseases, offer tremendous potential to gain insight into the etiology and treatment of these disorders. In essence, HIOs are the gateway to the development of personalized medicine.

We established small- and large-intestinal HIOs from resident intestinal stem cells derived from 2 individuals diagnosed with CCS (termed CCS1 and CCS2). These unique cultures allowed us to investigate potential differences between the CCS and non-CCS intestinal epithelium and provided novel insight into the disease that has not been previously possible. We found that the differentiated CCS HIOs maintained a more proliferative phenotype compared with non-CCS HIOs, suggesting a potential link between excess proliferation and the clinical presentation of polyposis. The HIOs from CCS donors also exhibited increased serotonin (5HT) producing enteroendocrine cells and had striking similarities in their appearance and transcriptional profile to HIOs genetically engineered to produce increased numbers of enteroendocrine cells (31). The association of 5HT with proliferation was confirmed in both non-CCS and CCS organoids, suggesting that dysregulation of enteroendocrine function and excess local 5HT production may play an important role in intestinal epithelial proliferation and explain the polyposis and other clinical features of CCS. Additionally, this work demonstrates the power of HIOs in understanding and developing new therapeutic modalities and targets to treat rare GI diseases.

## Results

### CCS exhibits alterations in intestinal secretory cell composition.

**Figure 1:**
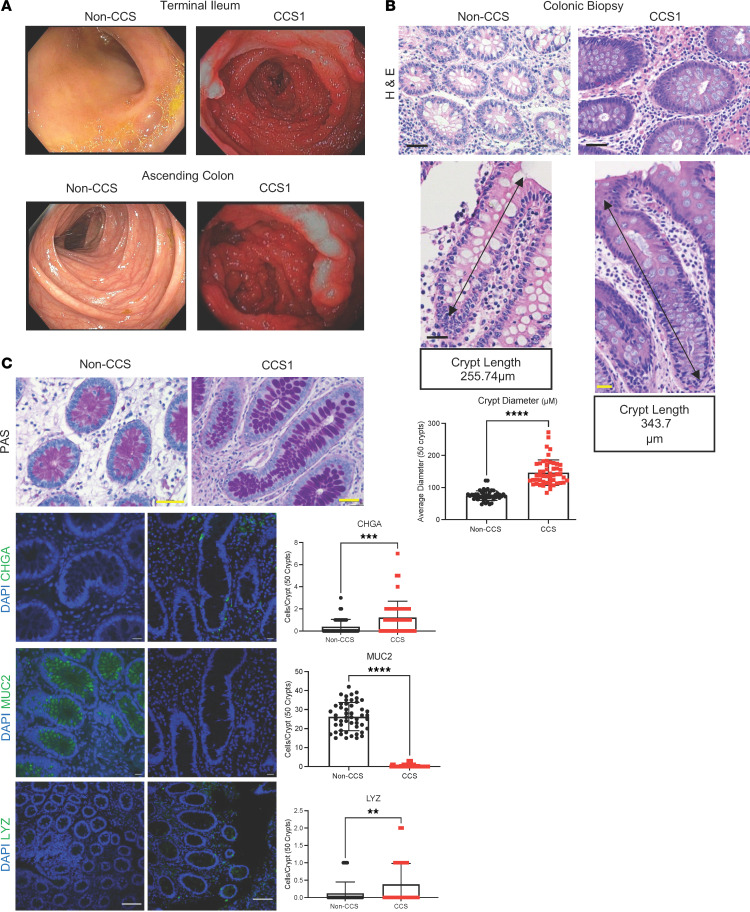
CCS1 exhibits alterations in intestinal secretory cell composition. (**A**) Endoscopic imaging revealed severe polyposis throughout the GI tract in CCS1. (**B**) Histological assessment of CCS1 and non-CCS colonic biopsies. Images were taken at ×40 magnification (scale bars: 25 μm). Crypt diameter was measured laterally across 50 crypts using the Nikon Imaging Software (*n* = 50). Longitudinal view of 1 representative crypt for CCS1 and non-CCS are shown with the measured crypt length (*n* = 1). (**C**) PAS staining for secretory cells and Immunostaining for EEC (CHGA), goblet cells (MUC2), (magnification, ×40, scale bars: 25 μm), and Paneth-like cells (LYZ) (magnification, ×20, scale bars: 100 μm) was performed on colonic sections from non-CCS or CCS1 tissue (green) and DAPI (blue). Quantification was performed by counting the number of positive cells per crypt for 50 crypts (*n* = 50). Bars show average number of positive cells mean ± SD. ****P < 0.0001; ***P < 0.0005, **P < 0.005, determined using a *t* test with Welch’s correction.

Endoscopic visualization of the GI tract of Patient 1 with CCS, also referred to as CCS1 (Figure 1A and Supplemental Figure 1A; supplemental material available online with this article; https://doi.org/10.1172/JCI166884DS1), and Patient 2 with CCS, also referred to as CCS2 (Supplemental Figure 2A), revealed the presence of hundreds of pedunculated polyps in the stomach, duodenum, ileum, and colon that are a defining feature of CCS (2, 7, 9, 12, 14). Histological assessment of biopsy specimens from the small and large intestine showed no dysplasia or adenomas consistent with the pathological data on CCS. Significant crypt and gland dilation was observed in both patients (Figure 1B and Supplemental Figure 2B) along with eosinophilic infiltration and active inflammation, confirming previous reports of crypt expansion in the intestines of patients with CCS (12–14). The average crypt diameter in the CCS colonic biopsies was measured laterally across crypts using Nikon software in H&E stained sections (Figure 1B and Supplemental Figure 2B) and was found to be over twice the diameter present in a non-CCS colonic biopsy. Methodology for measuring the crypt diameter is shown in Supplemental Figure 1C. Additionally, to show crypt depth, one representative image was taken for non-CCS, CCS1, and CCS2 to show that the CCS crypts have longer lengths compared with non-CCS crypts (Figure 1B and Supplemental Figure 2B). The average colonic crypt diameter measured in the H&E sections from CCS1 and CCS2 was compared to that obtained from patients with familial polyposis (FAP), a rare hereditary genetic condition that is associated with numerous precancerous adenomas in the large intestine (32–34). Colonic crypt diameter in biopsies from patients with FAP was not significantly enlarged compared with colonic crypt diameter in non-CCS biopsies (Supplemental Figure 3, A–C) or biopsies from CCS1 or CCS2 in a direct comparison (Supplemental Figure 3C), indicating that enlarged crypts are not an ubiquitous feature of polyposis disorders, but might be specific to CCS disease.

Further microscopic analysis using periodic acid-schiff (PAS) staining, which detects glycans common to secretory cells, suggested that colon biopsy specimens from patients with CCS had an increased abundance of secretory cells within the crypts (Figure 1C). To determine whether there were increases in specific subtypes of secretory cells, biopsy sections were incubated with antibodies against 3 known large intestinal secretory cell markers, CHGA (enteroendocrine cells), MUC2 (goblet cells), and LYZ (paneth-like cells) (35), followed by immunofluorescent imaging and quantification (Figure 1C and Supplemental Figure 2C). A 2-fold increase in the number of epithelial cells positive for CHGA (CCS1/2) and LYZ (CCS1) was seen in the biopsies from the patients with CCS compared with the non-CCS biopsy (Figure 1C and Supplemental Figure 2C). These specific increases in secretory cells were not observed in biopsies (*n* = 3) from patients with FAP (Supplemental Figure 3D). Our observations suggest that the crypt dilation observed in CCS intestinal biopsies may be associated with a specific expansion of secretory cells.

### CCS HIOs recapitulate features of CCS biopsies.

**Figure 2:**
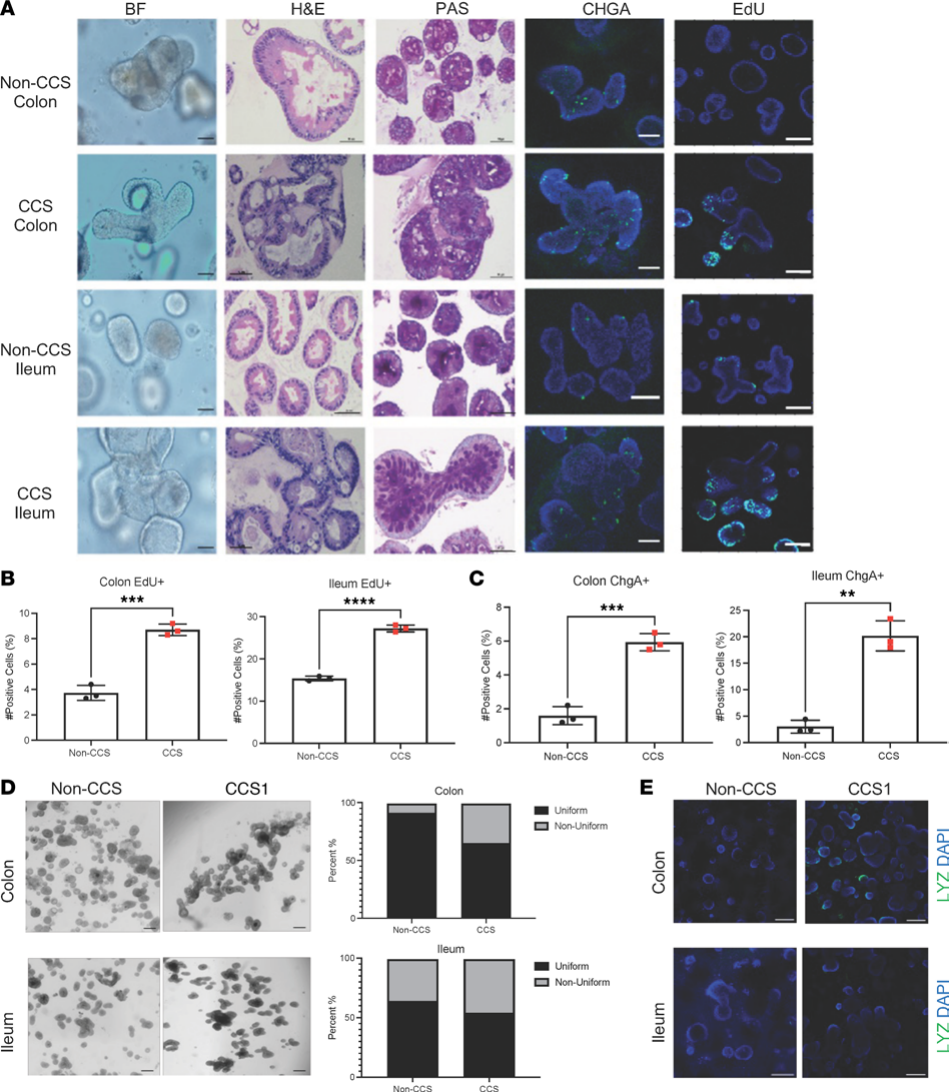
CCS1 HIOs are more proliferative and have increased EECs. (**A**) CCS1 and non-CCS HIOs morphology was assessed using bright field (BF), H&E, and PAS staining. Numbers of EECs were assessed with an anti-chromogranin A (CHGA) antibody (green). Proliferation was assessed by pulsing the HIOs with EdU and detecting uptake using the ClickIt EdU Imaging Kit (green) (*n* = 3). Images were taken at ×20 magnification (scale bars: 100 μm). (**B** and **C**) Flow cytometric detection of CHGA+ and EdU+ cells in each culture. Bars represent mean ± SD (*n* = 3). (**D**) Bright field image of non-CCS and CCS1 organoids. Images were taken at ×4 magnification (scale bars: 200 μm). Graphs quantitate percentage of HIOs in each culture with a uniform (less than 2 buds) and nonuniform morphology (3 or more buds) from the number of organoids within each well. (**E**) CCS1 and non-CCS HIOs were stained with LYZ (green) to mark Paneth-like cells. Images were taken at ×10 magnification (scale bars: 50 μm). ****P < 0.0001; ***P < 0.0005; **P < 0.005, determined using a *t* test with Welch’s correction.

HIOs were generated from biopsies obtained from the ileum and colons of CCS1 and CCS2, differentiated, and compared with differentiated HIOs established from biopsies from donors without CCS. Under bright field microscopy, the differentiated HIOs from the patients with CCS exhibited unique budding morphologies that were also seen by H&E staining compared with non-CCS HIOs (*n* = 3) (Figure 2A and Supplemental Figure 2D). HIOs were further quantified by their uniformity where the percentage of nonuniform HIOs with 3 or more buds was compared against uniform HIOs, with 2 or fewer buds. We found that CCS HIOs had more nonuniform organoids compared with controls (Figure 2B and Supplemental Figure 2E). PAS staining of the CCS1 HIOs indicated an increase in the abundance of polysaccharides characteristic of epithelial secretory cell lineages consistent with the increase seen in patient tissue (Figure 2A). In support of the alterations in secretory cells that associated with CCS in patient biopsy observations, differentiated CCS HIOs exhibited increased numbers of CHGA (Figure 2, A and D and Supplemental Figure 2D) and LYZ (Figure 2E) expressing cells. Based on these findings, the CCS HIOs appear to model histological aspects of the in vivo disease and could provide a deeper understanding of disease etiology.

In culturing both HIOs from patients with CCS, it was noted that the colonic and ileal-differentiated HIOs seemed to proliferate in the absence of growth factors compared with the non-CCS donor HIOs derived from the same intestinal segments. To quantify proliferative cells, we assessed cell division using 24 hour EdU incorporation and detection. We found more proliferating cells in the differentiated CCS HIOs compared with the non-CCS HIOs (Figure 2A, Supplemental Figure 2D). Quantification of EdU+ cells by flow cytometric analysis revealed CCS HIOs had significantly higher numbers of proliferating cells (*P* < 0.05) compared with non-CCS donor HIOs (Figure 2C, Supplemental Figure 2F). Therefore, HIOs from patients with CCS retain a high proliferative capacity in the absence of growth factors and have increased secretory cells, corresponding to observations in the patient tissue and further supporting a link between secretory cells and proliferation.

### Modification of HIOs resulting in increased enteroendocrine cells exhibit a CCS HIO phenotype.

**Figure 3:**
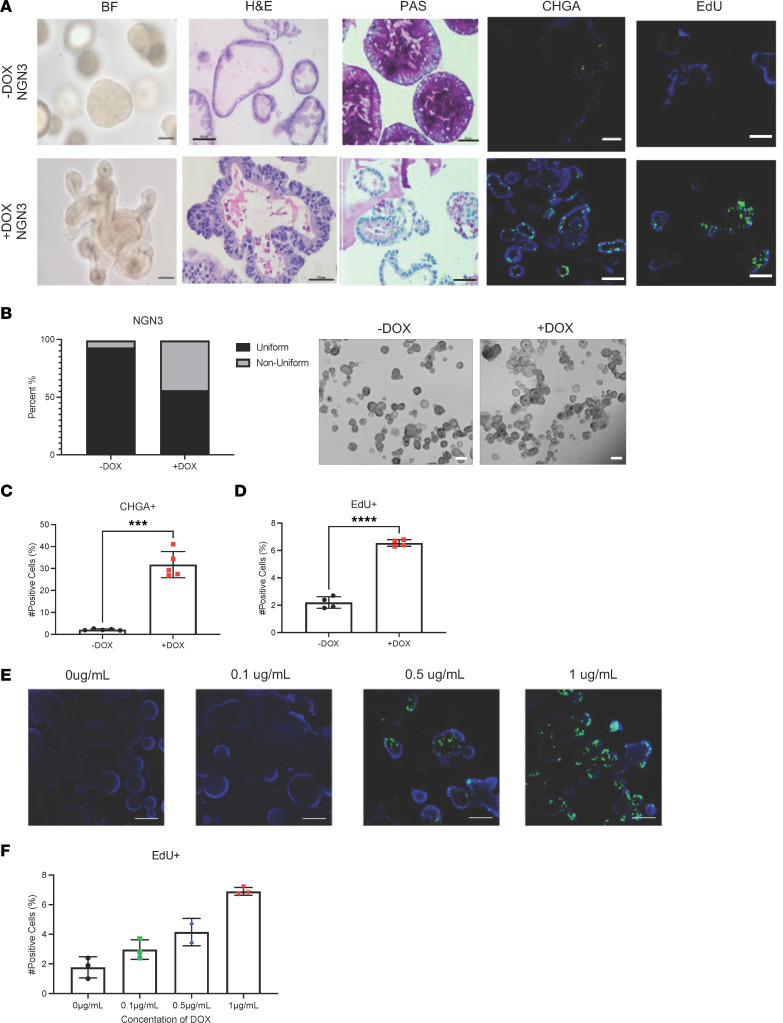
Modification of HIOs resulting in increased EECs exhibit a CCS HIO phenotype. (**A**) Bright field (BF), H&E, and PAS staining indicate unique morphology and increased secretory cells. Immunostaining for chromogranin A (CHGA), green cells, confirms induction. Nuclei were stained with DAPI (blue). Proliferation was assessed by EdU uptake and detected using ClickIt technology. Images were taken at ×20 magnification (scale bars: 100 μm) (*n* = 3). (**B**) Percentage of HIOs in each culture with a uniform (less than 2 buds) and nonuniform morphology (3 or more buds) from the number of organoids within each well. Images were taken at ×4 magnification (scale bars: 200 μm). (**C** and **D**) Flow cytometric detection quantified CHGA+ and EdU+ cells in each culture. Bars represent mean ± SD (*n* = 3). (**E**) HIOs were treated with increasing doses of doxycycline to induce increasing numbers of EECs. Proliferation was assessed using the ClickIt Edu Imaging Kit (green). Images were taken at ×20 magnification (scale bars: 100 μm). (**F**) Flow cytometric detection of EdU+ cells in each culture. Bars represent mean ± SD (*n* = 3) ****P < 0.0001, ***P < 0.0005 were determined using a *t* test with Welch’s correction.

Several studies implicate a role for enteroendocrine cells and their products in modulation of ISC proliferation (36–39). The finding of increased enteroendocrine cells and proliferation observed under conditions that typically result in differentiation in CCS HIOs suggested a potential link between the 2 observations. Recent work from our group used lentivirus transduction to inducibly express NGN3, a transcription factor required for the differentiation of enteroendocrine cells in human jejunal HIOs (NGN3-HIOs). It was previously reported that these NGN3-HIOs express increased enteroendocrine cells upon doxycycline-dependent induction (31). Bright field examination showed that NGN3-HIOs show similar unique budding morphologies and structures to those observed in the CCS HIOs (Figure 3, A and B) and different to uninduced or non-genetically modified HIOs (Supplemental Figure 4A). Immunofluorescent and flow cytometric detection of CHGA confirmed the increases in enteroendocrine cells reported in the induced NGN3-HIOs (Figure 3, A and C).

To assess whether NGN3-HIOs exhibited increased proliferative cells, EdU incorporation was used. Detection of EdU+ cells by both microscopy and flow cytometry indicated that overexpression of NGN3 resulted in increased numbers of proliferative cells compared with either uninduced NGN3-HIOs or WT HIOs treated with doxycycline (Figure 3, A and D and Supplemental Figure 4B). These results further support an association between increased enteroendocrine cells and HIO proliferation. Manipulation of doxycycline concentrations increased the enteroendocrine cell population in a dose-dependent manner (31). Treatment of NGN3-HIOs with increasing doses of doxycycline resulted in increased numbers of enteroendocrine cells that directly associated with an increase in proliferating cells (Figure 3, E and F) providing further evidence of a link between increases in enteroendocrine cells and HIO proliferation.

Intestinal stem cells and transit amplifying cells are thought to proliferate to populate the intestinal epithelium with terminally differentiated absorptive and secretory cells. To rule out the possibility that the increase in proliferating cells was solely due to proliferative enteroendocrine cells, NGN3-HIOs and CCS1 HIOs were dually stained for EdU incorporation and CHGA expression. CHGA+ cells were not found to be EdU+, but instead appeared to be adjacent to proliferating cells (Supplemental Figure 4, C and D), indicating that the enteroendocrine cell is not proliferating in the NGN3-HIOs or CCS HIOs. CCS1 and NGN3 HIOs did not stain with antibodies against POU2F3 (tuft cells) (Supplemental Figure 5). Additional goblet cell markers were also assessed in CCS HIOs (MUC2, MUC5B, and MUC5AC) (Supplemental Figure 5). Results matched the transcriptional data (Supplemental Figure 6) with high levels of MUC5B and lower levels of MUC2. The MUC2 staining in the HIOs was lower than in controls, recapitulating the findings of the in vivo tissue.

### CCS and NGN3-HIOs have similar transcriptional profiles.

**Figure 4:**
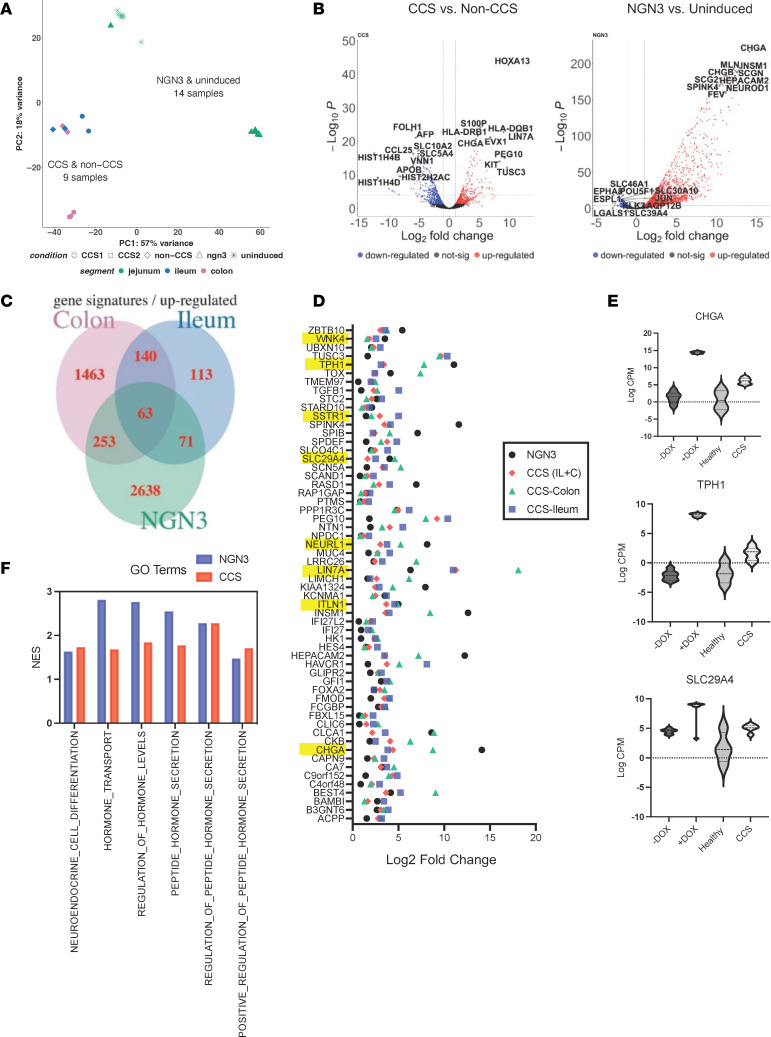
CCS and NGN3-HIOs have similar transcriptional profiles. (**A**) PCA of normalized log_2_ counts per million expression of CCS HIOs (*n* = 9), NGN3-HIOs (*n* = 14), and non-CCS and uninduced NGN3 HIOs [31]. (**B**) The number of differentially up regulated (Fold Change > 1.5) and down regulated (Fold Change < 1.5) genes between the data sets compared with their controls with an FDR ≤ 0.05. (**C**) Venn diagram shows the overlapping differentially expressed genes between CCS and NGN3 data sets. (**D**) 63 genes were upregulated (log_2_ FC) in CCS Colon (green), CCS Ileum (blue), CCS combined (red), and NGN3 (black) HIOs. Serotonin related genes are highlighted in yellow. (**E**) Log CPM values of serotonin related genes upregulated in NGN3 and CCS HIOs compared with their respective controls. (**F**) Normalized enrichment scores (NES) of hormone related pathways in the NGN3 and CCS HIOs compared with their controls with an FDR adjusted *P* value ≤ 0.25.

To gain deeper insight into differences in the CCS HIOs that might explain the etiology of the disease, transcriptional profiling was performed on CCS and NGN3- HIOs (31) and compared with transcriptional signatures from control HIOs. Principal component analysis (PCA) showed that the CCS and NGN3-HIOs were transcriptionally distinct from controls (18% variance), and the 2 organoid lines from patients with CCS (2 replicates from CCS1 and 1 from CCS2) clustered more similarly with NGN3-HIOs compared with noninduced NGN3 HIOs (Figure 4A). Volcano plots of the differentially expressed genes in the ileal CCS1 and jejunal NGN3-HIOs compared with their respective controls showed the expected increased expression of CHGA and other enteroendocrine markers with an FDR-adjusted *P* value under 0.05 (Figure 4B), consistent with the microscopy and flow cytometric data (Figure 2, A and C and Figure 3, A and C). Expression of identity gene sets associated with specific small intestinal epithelial cell populations (goblet, paneth, enterocyte, and stem cells) also showed consistent trends in genes between the 2 HIO models, CCS1 and NGN3 (Supplemental Figure 6), confirming the overall common increase in markers associated with secretory cell lineages. A decrease in the enterocyte markers in both HIOs was also observed (Supplemental Figure 6). Gene set enrichment analysis (GSEA) (FDR-adjusted *P* value < 0.25) performed on the differentially expressed genes in the CCS and NGN3-HIOs revealed enrichment of pathways related to neuronal, hormonal, and GPCR signaling, consistent with increased enteroendocrine cell numbers and function (Supplemental Figure 7A). As might be predicted by the decreased known transcriptional markers for enterocytes (Supplemental Figure 6), pathways involved in functional aspects of enterocytes, such as absorption, were decreased compared with their respective controls (Supplemental Figure 7A). Transcriptionally, the CCS and NGN3-HIOs appear to have a globally similar profile consistent with the similarities in physical properties.

We next examined the upregulated genes in common between the CCS and NGN3-HIOs to identify pathways that might link the increase in enteroendocrine cells with proliferation. There were 63 significantly upregulated transcripts in common with the NGN3 HIOs, the 2 colonic CCS1 and 2 HIOs, and the ileal CCS1 HIOs (Figure 4, C and D). Enriched in these common transcripts were several related to 5HT, a product produced by enteroendocrine cells. 5HT has many functions, including links to cell proliferation (37, 39–42). *SLC9A4*, a 5HT transporter, and *TPH1*, an important rate-limiting step in the synthesis of 5HT, were among the top common upregulated transcripts in both the NGN3 and the CCS HIOs (Figure 4, D and E). Other transcripts present in the common 63 genes (*CHGA, CLIC6, MUC2, LIN7A, SPINK4, SSTR1, TGFB1,* and *TUSC3*) were also indirectly linked to 5HT (Figure 4D). A more complete list of 5HT-related genes in CCS and NGN3-HIOs is described in Supplemental Figure 7B. The transcriptional findings suggest a link between increased enteroendocrine cells, 5HT production, and increased epithelial proliferation. Other studies have linked neuronal 5HT to proliferation in other cell types; however, (39, 41–43), dysregulation of 5HT as the cause of intestinal polyposis and proliferation has not been explored. GSEA pathway analysis confirmed the 5HT hypothesis with specific pathways upregulated for hormone signaling using an FDR-adjusted *P* value under 0.25 to generate the NES scores (Figure 4F). 

### CCS HIOs and patient biopsies demonstrate increased 5HT production.

**Figure 5:**
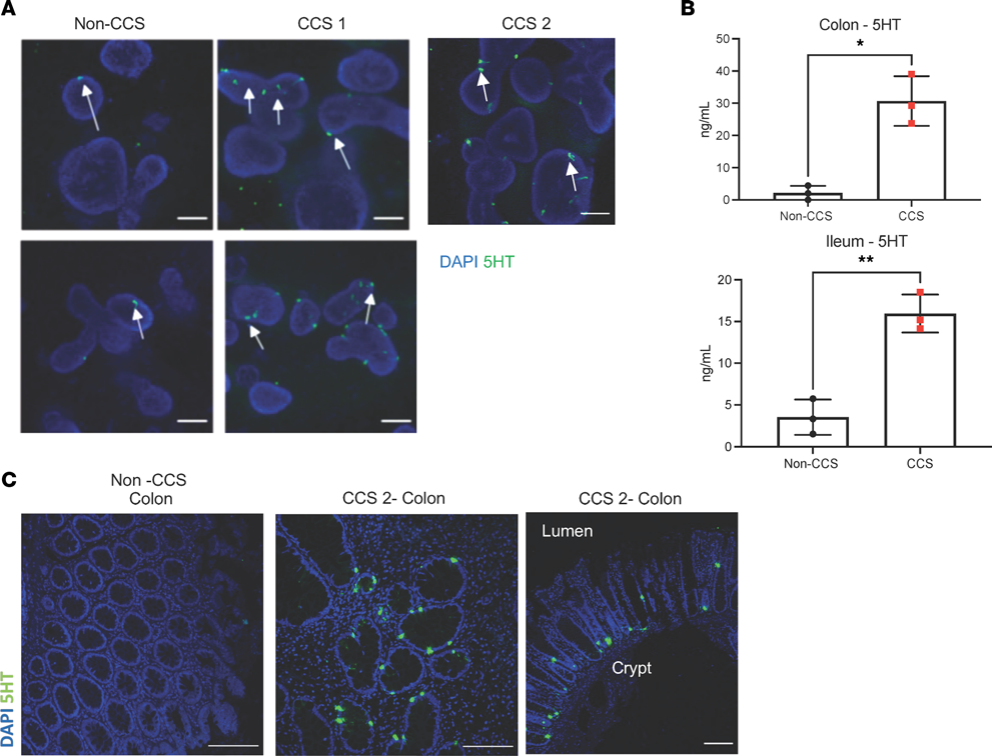
CCS1 HIOs and CCS2 patient biopsies demonstrate increased 5HT production. (**A**) CCS1 and 2 and non-CCS HIOs were stained with 5HT antibody (green) and DAPI (blue) (*n* = 3). Arrows indicate location of 5HT positive cells. Images were taken at ×20 magnification (scale bars: 100 μm). (**B**) Secreted 5HT in the media of the HIOs (CCS1) was measured utilizing a serotonin ELISA kit (Eagle Biosciences) and compared with non-CCS HIOs. Bars represent mean ± SD (*n* = 3). **P < 0.005, *P < 0.05 determined using a *t* test with Welch’s correction. (**C**) CCS2 patient tissue exhibits increases in 5HT matching the observations of the HIOs (*n* = 3). Images were taken at ×20 (scale bars: 100 μm) and magnification, ×10 (Scale bars: 50 μm).

The transcriptional analysis of the CCS and NGN3-HIOs suggested 5HT production associated with increased proliferation in the HIOs. To assess 5HT production in the CCS HIOs, we stained the CCS HIOs with an anti-5HT antibody (Figure 5A), and secreted 5HT levels were quantified in the culture media by ELISA (Figure 5B). Both the CCS1 and 2 colon and CCS1 ileum HIOs had increased numbers of 5HT+ cells compared with non-CCS HIOs. No staining was observed with antibodies against PYY and SST in CCS1, other known products for enteroendocrine cells. Consistent with the immunofluorescence results, culture media from CCS1 HIOs contained significantly higher levels of secreted 5HT compared with non-CCS donor HIOs (Figures 5, A and B). To confirm that 5HT was also upregulated within tissues from patients with CCS, paraffin embedded sections were stained with an anti-5HT antibody. Tissues from patient 2 with CCS showed an increase in the number of 5HT+ cells compared with a non-CCS control (Figure 5C). Further, cross sections of crypts from patient 2 with CCS show the 5HT+ cells located near the base of the crypt (Figure 5C, right panel), solidifying conceptually the potential geographic relationship between cells producing 5HT and the ISC. This work demonstrates that 5HT can regulate the proliferation of the intestinal epithelium and suggests that its dysregulated production by increased enteroendocrine cells might explain aspects of the etiology of CCS.

### 5HT associates with increased HIO proliferation.

**Figure 6:**
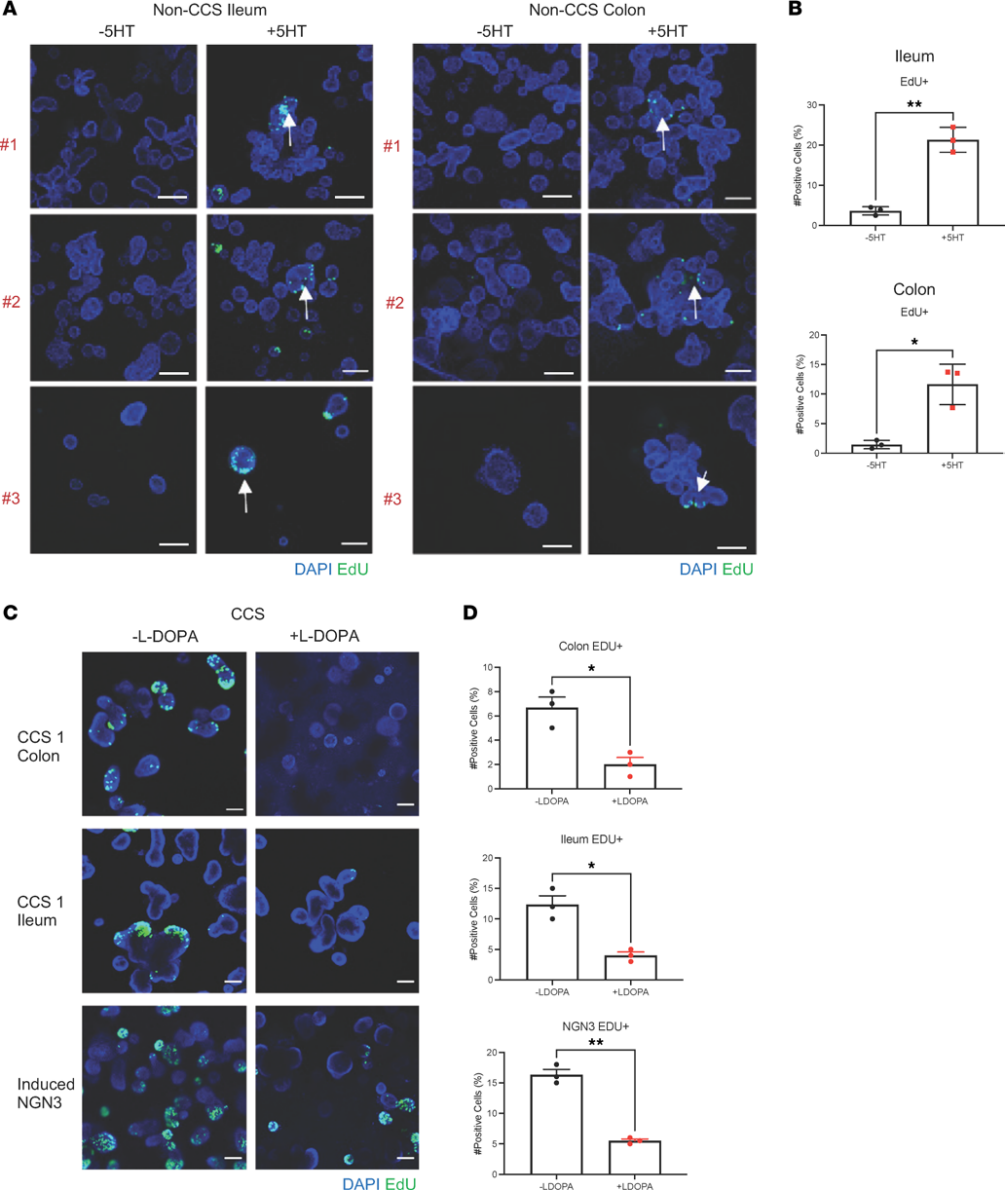
5HT associates with increased HIO proliferation. (**A**) 6 non-CCS HIO lines, 3 from the ileum and 3 from the colon, were treated with 600 ng of 5HT and proliferating cells were assessed using the ClickIt EdU Imaging Kit (green). Arrows indicate location of 5HT positive cells. Images were taken at ×10 magnification (scale bars: 50 μm) (*n* = 3). (**B**) Flow cytometric of EdU+ cells. Bars represent mean ± SD (*n* = 3). (**C**) Serotonin was inhibited with L-DOPA in CCS1 and NGN3 HIOs and proliferation was assessed using the ClickIt Edu Imaging Kit (green). Images were taken at ×10 magnification. (**D**) Flow cytometric of EDU+ cells. Bars represent mean ± SD (*n* = 3). ****P < 0.0001, **P < 0.005, *P < 0.05. Determined using a *t* test with Welch’s correction.

To directly test whether exogenous addition of 5HT to non-CCS HIOs could induce proliferation, exogenous 5HT was added to the media and matrigel of 6 differentiated non-CCS HIOs. Proliferation was induced in 3 independent ileal and colonic non-CCS HIOs by the addition of 5HT and increases in proliferation could be observed microscopically (Figure 6A) and quantified by flow cytometry (Figure 6B). To demonstrate that 5HT was essential for the proliferative capacity of the CCS HIOs, we inhibited 5HT synthesis with L-DOPA, a tryptophan hydroxylase (TPH1) inhibitor (44), which is the necessary enzyme for conversion of tryptophan to 5HT (45). L-DOPA was added to the cultures during differentiation and proliferation, and 5HT was assessed microscopically and quantified by flow cytometry (Figure 6, C and D and Supplemental Figure 8A). Treatment with L-DOPA did not change the MUC5B expression pattern (Supplemental Figure 8). Addition of the inhibitor resulted in abrogation of proliferation in the CCS1 HIOs, returning them to non-CCS HIO levels (Figure 6, C and D). These findings link 5HT to intestinal epithelial proliferation.

## Discussion

Affecting over 350 million people worldwide (Eurordis.org), rare diseases cumulatively affect 8%–10% of the population. Despite these staggering numbers, less than 10% of patients who suffer from a rare disease have a viable treatment or therapeutic option (46). This is due, in part, to a reliance on traditional research models of transformed cell lines and animal models, both of which, in many cases, do not accurately reflect the pathogenesis of many rare diseases. The ability to propagate human stem cells indefinitely in vitro and differentiate them into functional human epithelium (18, 20) has changed the landscape of research in rare human disease by overcoming some of the limitations of traditional models. These cultures, termed organoids, allow expansion of diseased epithelium from a stem cells derived from the organ of interest and allow the reconstruction of many features of the disease within the context of the individual genetic makeup, while offering the ability to gain new and mechanistic insight. Human organoid epithelial models have been successfully used to study several GI monogenic-based disorders, such as cystic fibrosis (47), microvillus inclusion disease (48), multiple intestinal atresia (49), and aberrant lipid metabolism, (50) and serve as an example of the use of organoid technology at the bedside. However, using organoids to model late onset rare disorders lacking a connection to a genetic mutation have proven to be more challenging. Organoids have been used to model less rare disorders such as celiac (51), inflammatory bowel disease (52, 53), and *Helicobacter pylori* infection (54). Here, we use patient tissue stem cell–derived intestinal organoids from individuals diagnosed with CCS, a rare disease typified by late-in-life onset, to demonstrate that the intestinal epithelium in these patients is characterized by increased enteroendocrine cells that produce 5HT. Furthermore, we show that 5HT induces cellular proliferation within the intestinal epithelium and offer a potential explanation for the clinical presentation of nonadenomatous cystic polyps. We confirmed these findings by genetically modifying organoids to inducibly express NGN3, a critical transcription factor necessary for enteroendocrine cell fate specification, and demonstrating an association with increased proliferation. This work illustrates the power of organoid cultures in the acceleration of insights into the etiology of rare diseases and is an example of how research results from the laboratory can be directly used to identify new ways to treat patients at the bedside.

Best known for its role as a neurotransmitter in the central nervous system, 5HT also functions as a hormone, regulating diverse biological processes such as mood, sleep, sexual function, and metabolism (55). Due to its pleiotropic effects, the 5HT pathway has served as a target for many therapeutic interventions designed to modulate these diverse pathways. Over 90% of 5HT in the body is produced by the enteroendocrine cells in the intestinal tract, where its role in motility has been well characterized (39–41). However, recent investigations implicate a role for 5HT in modulating epithelial homeostasis through the regulation of proliferation. Villus height, crypt diameter, and overall mucosal surface area are affected both by 5HT agonists and antagonists (56), suggesting that proliferative cells in the crypt, including the intestinal stem cell, may be a target for 5HT signaling. Other evidence supporting this relationship includes the preferential localization of TPH1-expressing enteroendocrine cells to the crypt region (57) and the expression of 5-HT receptors within the crypts (58). In intestinal tissue from CCS, we observe increased crypt diameter (Figure 1B), suggestive of increased proliferation that is replicated in the organoid model (Figure 2A) concurrently with the detection of increased 5HT positive enteroendocrine cells. By using an inducible genetically engineered organoid, which is characterized by increased enteroendocrine cells via overexpression of the necessary transcription factor, we demonstrate a correlation between numbers of enteroendocrine cells and the presence of increased proliferative cells (Figure 3). Conversely, inhibition of 5HT in the CCS organoids results in a significant reduction in proliferative cells (Figure 5), confirming that 5HT modulates intestinal epithelial proliferation and regeneration. The implications of this finding are wide ranging. With the arsenal of 5HT transport inhibitors and receptor agonists that are approved for use, there may be a new opportunity to exploit these therapeutic options for intestinal repair and regeneration in the context of injury or damage.

The finding that 5HT plays a role in the etiology of CCS is consistent with other clinical manifestations associated with the syndrome (Supplemental Figure 9), and, as such, provides a potential lead into events that may initiate the disease. Inflammation is thought to be a critical factor in CCS, but other intestinal inflammatory syndromes are not associated with polyp growth. Potentiation of 5HT signaling has been linked to an increase in proinflammatory cytokines (59), and the lineage specification of enteroendocrine cells from proliferating precursors can also be influenced by immunologic control (60). Additionally, blocking 5HT can reduce intestinal inflammation (61). It may be that the increased epithelial 5HT provokes the inflammation observed in the intestines of patients with CCS and provides a potential explanation for the failure of antiinflammatory and autoimmune therapies to treat this disease (62, 63). Crosstalk between serotonergic and cholinergic regulation (56) also suggests a role for 5HT in chronic diarrhea, which is a defining feature of CCS. 5HT has also been implicated in pathways that result in intestinal neoplasia (64, 65). Although the polyps that characterize CCS are nonadenomatous, the link between 5HT and proliferation in the organoids provides indirect evidence, supported by the association in neoplasia, that 5HT may be a critical factor in abnormal intestinal epithelial growth.

Though our current findings do not lead directly to this conclusion, we find it tantalizing to consider other manifestations of CCS and their potential link to 5HT. For example, niacin, a component of hair and nails, is synthesized from tryptophan in an alternative pathway to 5HT. Since tryptophan is required for niacin synthesis, it could be speculated that available tryptophan is being diverted to 5HT synthesis, limiting its availability for niacin production and resulting in the adverse effects on hair and nails observed in CCS. In a similar way, melatonin is a metabolite of 5HT, also synthesized from tryptophan in an alternative pathway to 5HT. Deficiencies in melatonin production result in sleep disturbances and poorly coupled circadian rhythms (66). Although not officially documented in CCS, both of the patients with CCS included in this work reported difficulty sleeping. Another clinical manifestation commonly seen in patients with CCS is hyperpigmentation of the skin. A role for 5HT in melanin production is less clear but there are links between 5HT levels and increased melanogenesis (67) that might also explain this clinical feature. Altered 5HT is a critical factor that plays a role in many mental illnesses (68). Although diagnosis of mental illness is complicated in patients with chronic GI diseases (69), both patients with CCS presented here had evidence of depression and anxiety. More work is necessary to understand the link between elevated local intestinal 5HT and non-GI manifestations and to test our working model of CCS (Supplemental Figure 9).

In conclusion, our studies used organoid technology to gain new insight into the etiology of CCS. We exploited advances in genome editing resulting in an engineered disease organoid to confirm and extend the impact of the use of organoids in understanding CCS by connecting aspects of the disease to cellular functions. The use of organoid technology revealed the potential of a novel therapeutic target for CCS, 5HT, whose efficacy in the treatment of CCS remains to be explored clinically. The association of NGN3 overexpression in the organoids with increased proliferation implies that dysregulation of NGN3 expression could explain the increased enteroendocrine cells in CCS. Due to the limited availability of biopsy tissue, we were only able to demonstrate increased 5HT expression in the patients with CCS. Questions still need to be addressed as to whether NGN3 expression is the initiating event that causes increased enteroendocrine cell production and thus plays a critical role in disease etiology. Organoid cultures may prove to be pivotal in evaluation of the role of NGN3 activation in CCS. Moving forward, increasing the complexity of epithelial organoids derived from patients with rare disease by the inclusion of stromal components such as immune, neuronal, and endothelial elements, will result in improved in vitro disease models that will allow deeper insight into rare clinical diseases at the cellular level In addition, the microbiome is a critical factor that must be considered in the etiology of the disease. 16S rRNA microbial profiling and composition of the microbiome was compared with healthy donors and patients with FAP and suggested alterations in diversity and composition (Supplemental Figure 10). Our findings support and extend those of Kim et al., who recently published a case report on the utilization of a fecal microbiome transplant to successfully treat a patient with CCS (70). More work is necessary to determine what role these alterations play in the disease and whether microbial composition affects or alters enteroendocrine cell function or 5HT production. These next generation organoids will continue to facilitate the detection, diagnosis, prognosis, and cures for rare diseases and will allow strong connections to continue to form between the beside and the bench.

## Methods

### Organoid establishment and propagation

#### CCS1.

A 38-year-old female of Asian (Laotian) descent presented with fatigue, dizziness, alopecia, dysgeusia, and weight loss. Physical exam revealed onychodystrophy and hyperpigmentation of the face and hands. Endoscopy revealed hundreds of erythematous, pedunculated polyps in the stomach, duodenum, and ileum while the colon had close to a dozen large pedunculated polyps. Polypectomy was performed with no diagnoses on histology. Germline genetic testing revealed no inherited polyposis syndromes. Stool multiplex PCR assay was negative for enteric microbial pathogens. A diagnosis of CCS was made. Currently the patient is maintained on azathioprine and antihistamines.

#### CCS2.

A 62-year-old white female (Greek descent) presented with abdominal pain, diarrhea, weight loss, hair and nail loss, and loss of smell and taste. Exam revealed a thin female with diffuse hair loss, including head, pubic area, and extremities, and dystrophic nails. Endoscopy revealed diffuse inflammation in the body and antrum of the stomach and the duodenum and diffuse inflammation in the colon with scattered polyps. Histopathology revealed hamartomatous polyp changes and eosinophilic predominant mixed inflammation consistent with CCS. She was treated initially with infliximab and azathioprine, but the azathioprine was stopped due to the development of abnormal liver functions tests, and so she was subsequently treated with monotherapy with infliximab.

Organoid cultures were derived from biopsies taken during endoscopy procedures from tissue adjacent to polyps in patients with CCS. Non-CCS organoids were obtained from individuals undergoing routine colonoscopy screenings. All HIOs were grown as 3D cultures in matrigel maintained by the Texas Medical Center Digestive Disease Center (TMC-DDC) GEMS Core, Baylor College of Medicine, as previously described (16, 17). Colonic biopsies were taken from the ascending colon. Ileum biopsies were taken from the terminal ileum. The Institutional Review Boards at Baylor College of Medicine and the University of Texas at Austin approved the study protocol to obtain tissue samples from which the organoid lines were established, and informed consent was obtained from both the patients.

Non-CCS organoid lines were chosen from the TMC-DDC catalog to approximately match the age and sex of the CCS organoid lines. HIOs were differentiated for 3 days by growing in the absence of growth factors from the media (WNT, RSPO, and Noggin). The NGN3 overexpressing line (NGN3-HIOs) was generated as described previously (31), and induction of enteroendocrine cells was performed by addition of 1 μg/mL doxycycline during differentiation. CCS and non-CCS organoid lines were differentiated for 3 days following previously published protocols (16–19).

### FAP patient samples

Tissue samples from patients with FAP were obtained from MD Anderson Cancer Center under an IRB approved protocol. Samples from patients with FAP were taken from nonpolyp tissue from the ascending colon and approximately age and sex matched to our CCS patients.

### Immunofluorescence and histology of tissue sections and organoids

Biopsy sections were fixed in 4% paraformaldehyde/PBS prior to paraffin embedding and sectioning. H&E and PAS staining was performed by the TMC Digestive Disease Center-Cell and Morphology Core histologist using standard protocols. Slides for IF were deparaffinized in histoclear and rehydrated with successive ethanol washes. Antigen retrieval was performed with 10 mM sodium citrate (71) for 1 minute in a pressure cooker and nonspecific binding was blocked with 1× PowerBlock (Biogenex). Slides were stained with primary antibodies overnight at 4°C, washed in PBST (0.05% Tween20/PBS), and incubated with secondary antibody at room temperature for 1 hour. Nuclei were detected by incubating in DAPI [NucBlue/PBS] (Thermo Fisher Scientific), 2 drops/mL for 15 minutes at room temperature. The number of cells positive for a specific antibody per crypt were counted in 50 randomly selected crypts across the stained slide section.

3D organoids were fixed with 4% (diluted from 16% stock concentration) paraformaldehyde (PFA) in PBS for 30 minutes at room temperature on a plate rocker. The fixative was quenched with 50 mM NH_4_Cl for 30 minutes at room temperature. Cells were permeabilized with 0.1% (500 μL/500 mL)Triton X 100 in PBS (Thermo Fisher Scientific) overnight at 4°C. Nonspecific binding was blocked using 3% (1 g/100 mL) BSA (Sigma-Aldrich) in PBS for 15 minutes. For EdU staining, we followed the manufacturer’s recommendation (Thermo Fisher Scientific). The 10 μM stock solution of EdU was diluted in PBS rather than DMSO to favor organoid health. The final concentration of EdU added to the cell culture media was 0.01 μM (72). EdU was pulsed for 24 hours.

For all other stains, cells were incubated with primary antibodies overnight at 4°C, washed in 3% BSA, and incubated with secondary antibody at room temperature for 1 hour. Nuclei were detected by incubating in DAPI for 15 minutes at room temperature.

CHGA Antibody (ImmunoStar, cat no. 20085) was used at a 1:1,000 dilution from a 1/10 concentration. MUC2 Antibody (SantaCruz, cat no. sc-515032) was used at a 1:500 dilution. LYZ Antibody (Thermo Fisher Scientific, cat no. PA1-29680) was used at a 1:500 dilution. MUC5B Antibody (Sigma-Aldrich, cat no. HPA008246) was used at a 1:500. MUC5AC (Thermo Fisher Scientific, cat no. MA5-1217) was used at a 1:500 dilution. 5HT Antibody (ImmunoStar, cat no. 20080) was used at a 1:1,000 dilution from a 1/10 concentration. Secondary antibodies from Jackson Laboratories included anti-rabbit Alexa-Fluor 488 (711-545-152), anti-mouse Alexa-Fluor 488 (715-545-150), and anti-rat Alexa-Fluor 488 (712-546-150) and were used at 1:1,000 dilutions.

For CHGA/EdU costained images, EdU staining was completed first following the manufacturer’s instructions (Thermo Fisher Scientific) followed by blocking using 3% BSA (Sigma-Aldrich) in PBS for 15 minutes followed by incubation with CHGA antibody overnight at 1:1,000 at 4°C, washed in 3% BSA, and incubated with secondary antibody at room temperature for 1 hour. Nuclei were detected by incubating in DAPI for 15 minutes at room temperature.

Bright field images were taking at either ×20 or ×40 magnification using a Nikon Ci-L bright field microscope and the associated software present in the Baylor College of Medicine Integrated Microscopy Core. Confocal immunofluorescence images were taken using the Nikon A1-Rs microscope and the corresponding software from the same core. Images were postprocessed using the ImageJ software.

### Flow cytometric quantification of proliferating and CHGA expressing cells

3D organoids were dissociated into single cells by first washing the cells out of the matrigel plug and incubating the cells in cold cell recovery solution (Corning) on ice for 10 minutes. Wells were spun at 400*g* for 3 minutes. Cells were dissociated using an accutase digestion reagent (Sigma-Aldrich) and incubated at 37°C for 30 minutes. Cells were disrupted every 10 minutes by gentle pipetting. After 30 minutes, the cells were pelleted (400*g* for 3 minutes) and resuspended in 1 mL of CMGF- media. This mixture was pipetted up and down 30 times quickly while avoiding air bubbles. Finally, the suspension of cells was passed through a 40 μm filter (VWR) and washed 1 time with 1% BSA/PBS prior to fixing.

Detection of EdU by flow cytometry was done using Click-It technology (Thermo Fisher Scientific). Cells were pulsed with EdU 24 hours before harvest. The 10 μM stock solution of EdU was diluted in PBS rather than DMSO to favor organoid health. The final concentration of EdU added to the cell culture media was 0.01 μM (72). The rest of the procedure was carried out using the manufacturer’s protocol.

For CHGA stained cells, after single cells were generated, cells were fixed in 4% PFA for 45 minutes on ice. Cells were washed using a 0.01% solution of PBS and Tween 20. Cells were blocked using a 5% donkey serum (Sigma-Aldrich) in 1% BSA for 20 minutes on ice. Primary CHGA antibody was added as a dilution of 1:1,000 in 1% BSA overnight at 4°C. After washing with 1% BSA 3 times for 5 minutes, secondary antibody, anti-rabbit (Thermo Fisher Scientific; A-21206) was added at a concentration of 1:5,000 in 1% BSA and incubated overnight at 4°C. Finally, the cells were washed 3 times with 1% BSA before analysis on the flow cytometer.

### Transcriptional and enrichment analysis

Transcriptional data sets included in this manuscript are submitted in GEO for public analysis under GSE207838. Total RNA-Seq analysis was performed on differentiated organoid cultures and samples from patients with FAP. Briefly, total RNA was extracted using the QIAshredder and Rneasy Mini Kit (Qiagen) with the inclusion of the optional Dnase digestion following the manufacturer’s instructions. Total RNA recovered was sent to Novagene Co. for library preparation (poly A enrinchment) and sequencing with NovaSeq PE150 (6G raw reads per sample). Raw sequence reads were checked for quality using FASTQC package ver. 0.11.9 and reads were trimmed with TrimGalore ver. 0.6.5 with default settings for adaptive trimming and for base quality filtering (73). Trimmed reads were aligned to human genome build GRCh38.98 using HiSAT2 ver 2.2.1 and a count matrix was generated from the aligned reads using featureCounts (74). Differential gene expression analysis was performed for the protein coding genes using the EdgeR ver. 3.32.1 R package (75). Significance was achieved for FDR-adjusted *P* value under 0.05 and a fold-change greater than 1.5. Enriched pathways for each comparison of interest were determined using GSEA v 3.3 using the Gene Ontology pathway compendium curated by the MsigDB database (76, 77). The GSEA analysis was performed on rank files using gene symbols and log_2_ fold changes based on the EdgeR analysis with significance achieved for FDR < 0.25.

### 5HT assays

5HT was detected in the organoid cultures using an anti 5HT antibody from Immunostar (cat no. 20080) at a 1:1,000 dilution. 3D organoids were fixed with 4% PFA in PBS for 30 minutes at room temperature on a plate rocker. The fixative was quenched with 50 mM NH_4_Cl for 30 minutes at room temperature. Cells were permeabilized with 0.1% Triton X 100 (Thermo Fisher Scientific) overnight at 4°C. Nonspecific binding was blocked using 3% BSA (Millipore) in PBS for 15 minutes. Cells were incubated with primary antibodies overnight at 4°C, washed in 3% BSA, and incubated with secondary antibody at room temperature for 1 hour. Nuclei were detected by incubating in DAPI for 15 min at room temperature.

5HT was detected in the media by enzyme-linked immunosorbent assay (Eagle Biosciences) according to the manufacturer’s instructions. A standard curve of known 5HT concentrations was plotted against optical density at 450 nm with a limit of detection of 2.6 ng/mL (Infinite F200Pro; Tecan).

Exogenous 5HT was added to 6 healthy non-CCS organoid lines (3 ileal and 3 colonic) that were selected from the TMC-DDC catalog. 600 ng/mL of 5HT was added to the matrigel at plating and then added daily to the media during the differentiation process for 3 days (Sigma-Aldrich). Cells were pulsed with EdU 24 hours before organoid harvest.

5HT was inhibited using L-DOPA, or 3,4-Dihydroxy-L-phenylalanine (Sigma-Aldrich). L-DOPA inhibits 5HT synthesis by blocking the function of TPH1, the critical enzyme for conversion of tryptophan to 5HT. L-DOPA was diluted to a concentration of 600 ng in 1 M Tris HCl (78) and was added to the matrigel at plating and the media every day for 3 days. Cells were pulsed with EdU 24 hours before organoid harvest.

### Microbial 16S analysis

16S rRNA microbial sequencing and analyses was performed on 3 replicates of stool samples from CCS1 (*n* = 3), stools from 19 patients with FAP (*n* = 19), and 19 stools from 19 healthy individuals by the Baylor College of Medicine Alkek Center for Metagenomics and Microbiome Research (CMMR) using their standard methods. Briefly, DNA was extracted utilizing the MagAttract PowerSoil DNA Isolation Kit (MO BIO Laboratories) and immediately stored at –80°C before the amplification step. Amplification was completed on the V4 hypervariable region of the 16S rRNA gene. The V4 region was amplified by PCR using primers 515F (GTGCCAGCMGCCGCGGTAA) and 806R (GGACTACHVGGGTWTCTAAT) and sequenced in the MiSeq platform (Illumina) (79–81). This protocol targets at least 10,000 reads per sample. We used the developed pipeline at the CMMR for the bioinformatics analysis. Briefly, the read pairs were demultiplexed based on the unique molecular barcodes added via PCR during library generation, then merged with bbmerge.sh (BBMap version 38.82), with merge parameters optimized for the 16SV4 amplicon type (maxstrict=t qtrim=t trimq=15). Merged reads were then further filtered using vsearch (82), allowing for a maximum expected error of 0.05, maximum length of 254 bp and minimum length of 252 bp. All the reads are combined into a single fasta file for further processing. Sequences were assigned to operational taxonomic units (OTUs) at a similarity cutoff value of 97%. The OTUs were subsequently mapped to an optimized version of the SILVA database to determine taxonomies (83, 84). The data sorted at the genera level were used to pick genera that were of greater abundance in CCS (Supplemental Figure 8C) and lesser in CCS (Supplemental Figure 8D) compared to patients with FAP and individuals in the control group.

### Statistics

While data from a single representative experiment is shown in immunofluorescence images, each experiment was performed 2 or more times. All other experiments were performed 3 times with at least 3 technical replicates per culture condition and time point. All statistical analyses were performed in GraphPad Prism Version 9.0 for Windows (GraphPad Software). Comparisons between groups was performed using an unpaired 2 tailed *t* test with Welch’s correction. *P* < 0.05 was considered to be statistically significant.

### Study approval

Study protocols were all approved by each institution via IRB committee. Written and informed consent was obtained from both patients prior to participation in obtaining the biopsy samples or stool samples. Records of informed consent have been retained by Baylor College of Medicine, Houston, Texas, MD Anderson Cancer Center, Houston, TX,and Dell Medical School, Austin, Texas.

### Data availability

The RNA-Seq data generated in this study are deposited in the Gene Expression Omnibus database: CCS and Non-CCS HIOs, GSE207838; NGN3 HIOs, GSE138350; and FAP and Healthy Patients, GSE153385, GSE156172, GSE106500. Codes are available from the corresponding author upon request. Raw data is available in an associated Supporting Data Values file.

## Author contributions

VP designed and performed experiments, analyzed data, prepared figures, and cowrote the manuscript. CB helped with experimental design and techniques and edited the manuscript. SCD and HAD provided data from the NGN3-induced HIOs, provided experimental advice, helped analyze data, and edited the manuscript. AK and XZ cultured all of the HIOs that were utilized to complete the experiments and gave experimental advice. HNP and CC analyzed the provided figures for the RNA-Seq experiments. EV and ND provided experimental advice, reagents, and generated the data from FAP patients. SM and HJK generated the HIO line from the second CCS patient and edited the manuscript. RS and LAF treated the CCS patients and provided us with the biopsy samples needed to generate the HIOs, provided tissue sections and images used to generate data and figures, and edited the manuscript. FM provided the 16S rRNA-Seq data for the FAP patients and edited the manuscript. MKE and RB provided conceptual advice, helped with experimental design, and edited the manuscript. SEB directed the project and cowrote the manuscript.

## Supplementary Material

Supplemental data

Supporting data values

## Version 1 11/01/2023

Electronic publication
