# Supplementary material for: Human intestinal organoids from Cronkhite-Canada syndrome patients reveal link between serotonin and proliferation
Source: J Clin Invest. 2023 Nov 1;133(21):e166884. doi: 10.1172/JCI166884 (PMC10617781; doi:10.1172/JCI166884)
Supplement: Supplemental data [file jci-133-166884-s065.pdf]

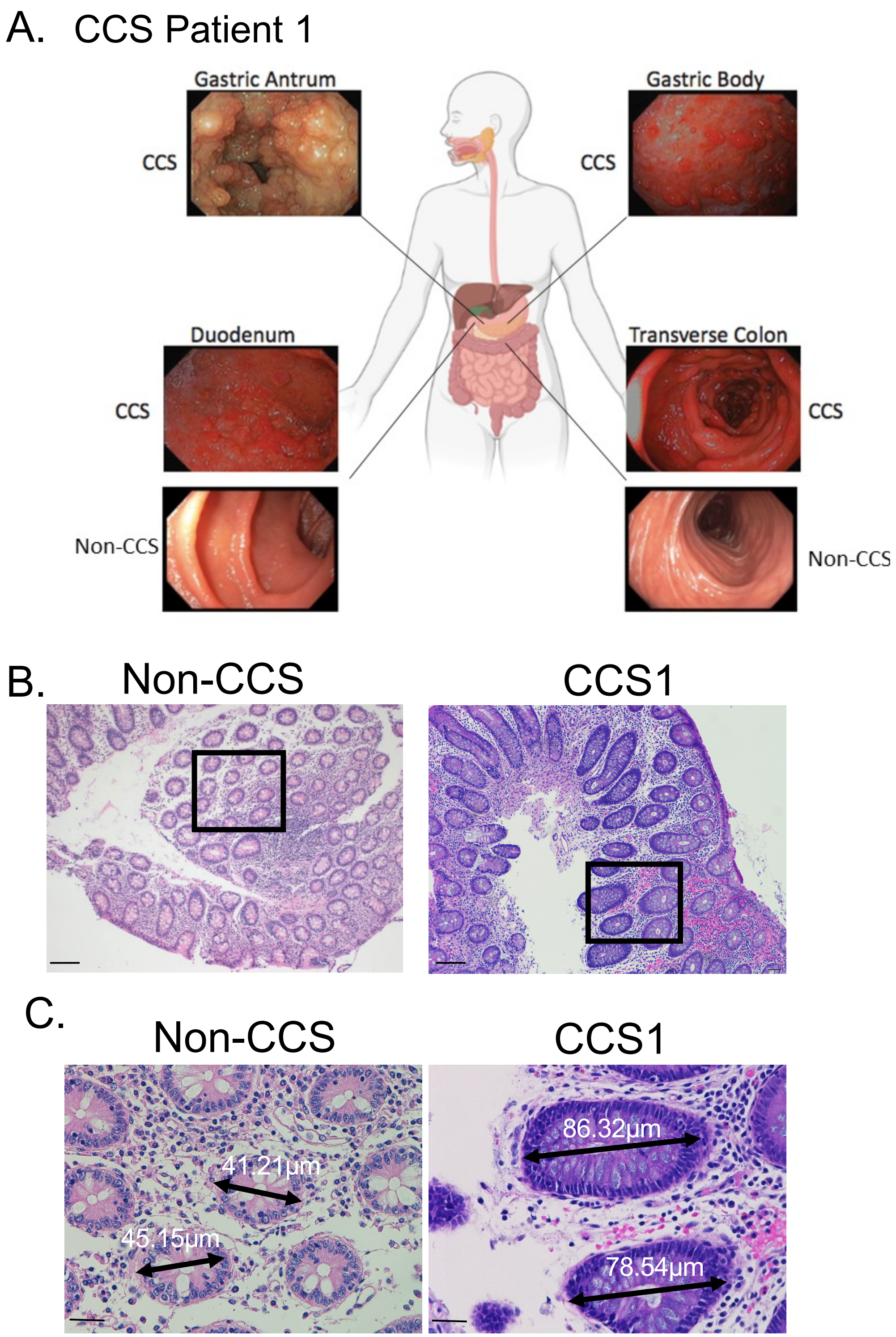

Supplementary Figure 1: Polyposis is present throughout the length of the intestinal tract in CCS Patient 1

(A) Additional endoscopic images highlight the severe polyposis throughout the GI tract in CCS patient 1 (CCS1). Polyps are present in the stomach, duodenum, and transverse colon. Duodenum and transverse colon are compared to a non-CCS control. Created with Biorender.com. (B) Zoomed out images of crypts from CCS1 and non-CCS (shown in Figure 1) to further show large crypts (4X, Scale = 50µm). Boxes indicate where images were taken for Figure 1B to ensure the same orientation. (C) Methodology to measure crypt diameter is demonstrated on a zoomed in image of Figure 1. 50 crypts were counted using the Nikon imaging software. Diameter was determined as left most basal side to right most basal side laterally across crypts.

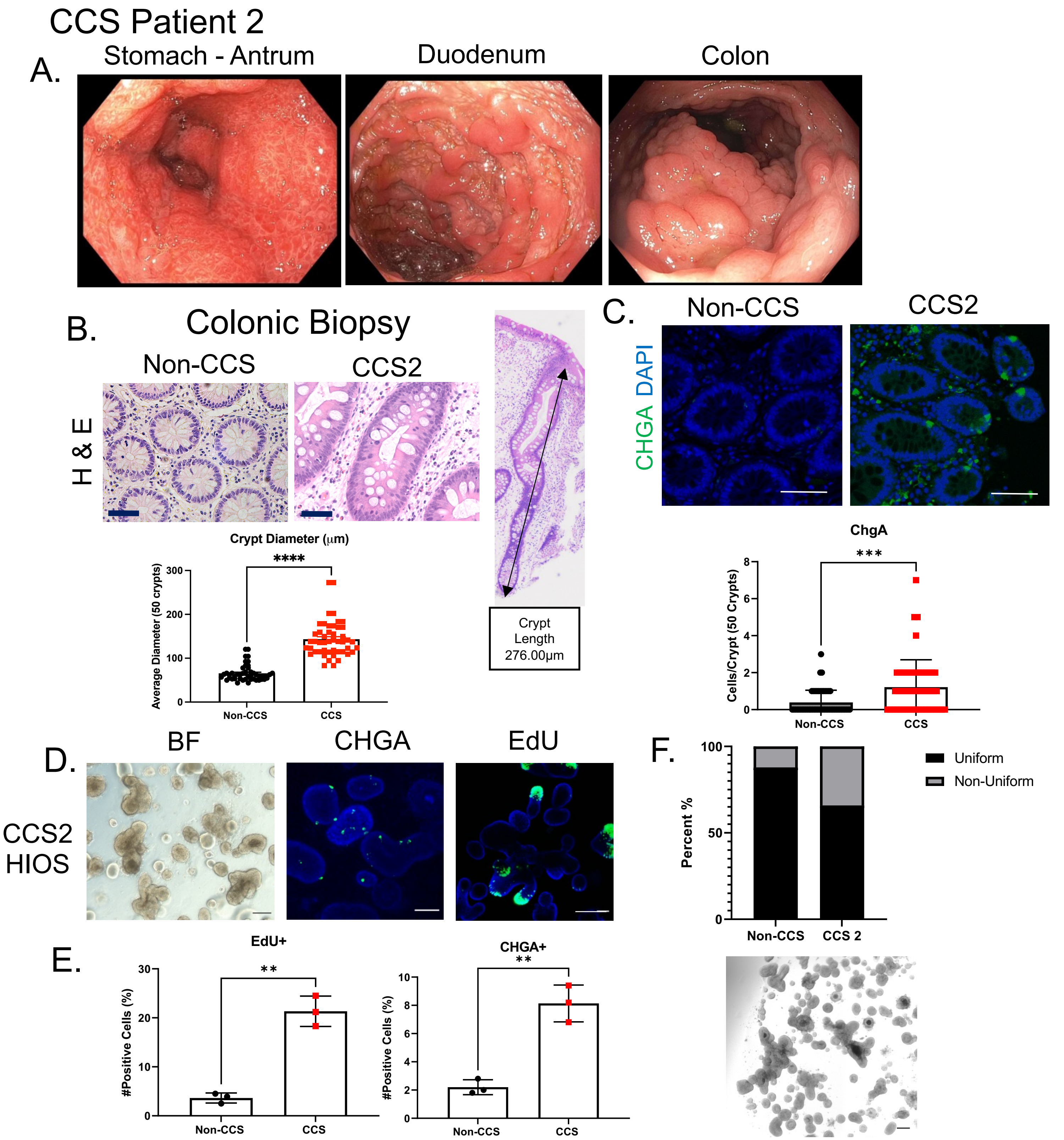

Supplementary Figure 2: Histology and HIOs from CCS Patient 2 mimics the characteristics observed from CCS Patient 1 (A) Endoscopic images of the intestine of CCS Patient 2 (CCS2). (B) Histological assessment of colonic biopsies from CCS patient 2 (CCS2) compared to a non-CCS control at 40X (Scale = 100μm). We measured the diameter of 50 crypts using the Nikon Imaging Software. We also assessed the crypt length of one representative longitudinal crypt (4X, scale = 50μm). (C) Immunostaining for enteroendocrine (CHGA) was performed on paraffin embedded colonic sections from CCS2 compared to a non-CCS control (GREEN). Nuclei were stained with DAPI (BLUE). Green cells were quantified by counting the number of positive cells in 50 crypts per section. Bars show average number of positive cells per field of view +SD. Images were taken at 40X (Scale = 100μm). (D) HIOs were generated from CCS2 colon and stained with EdU and CHGA (GREEN) and DAPI (BLUE). Images were taken at 20X (Scale = 150μm). (E) EdU+ and CHGA+ cells were assessed by flow cytometry. (F) Budding morphology in CCS HIOs were documented as a percent of the total HIOs per well. Bars represent mean + SD (n=3). ***, p<0.0005; **, p<0.005 determined using an unpaired two-tailed t-test with Welch's correction.

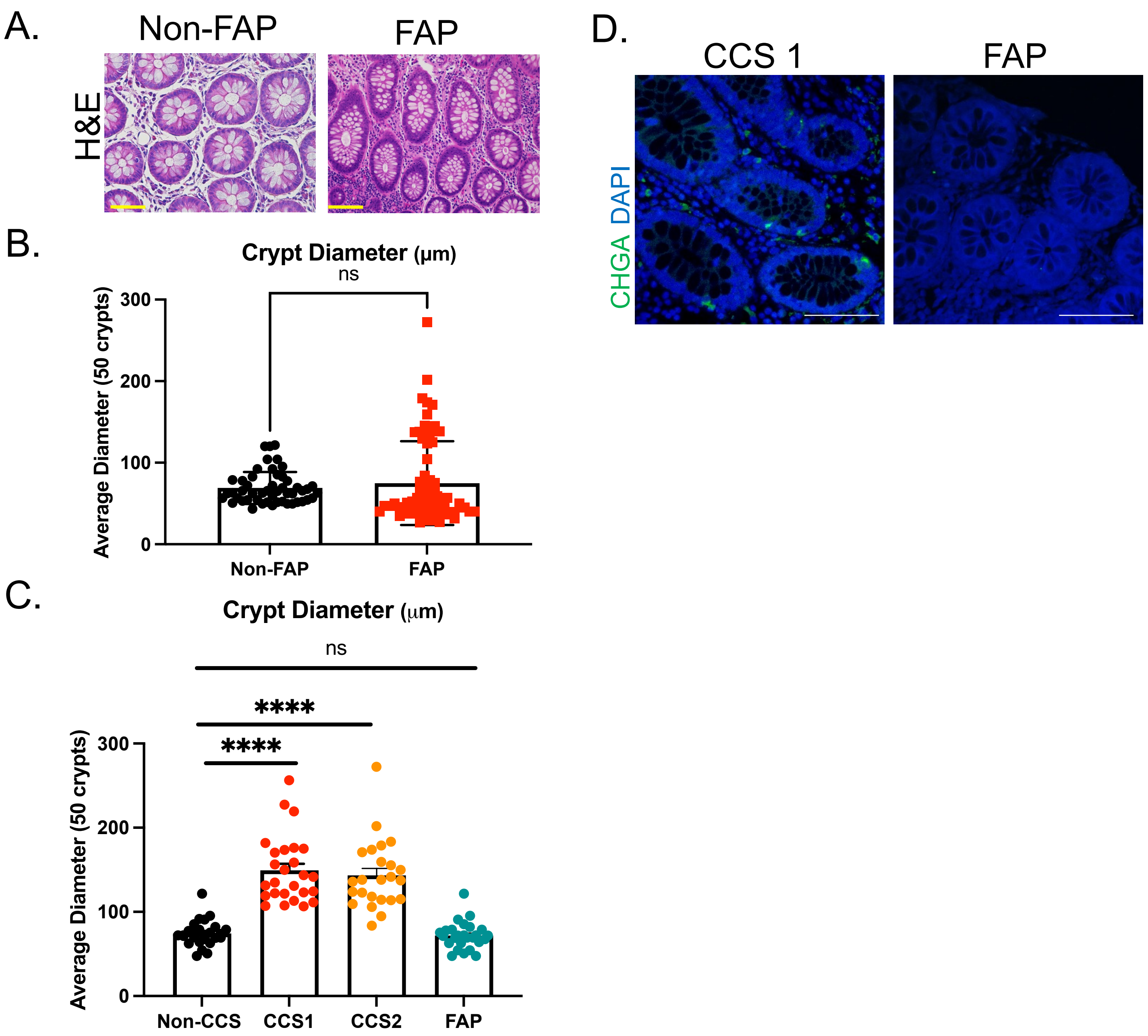

Supplementary Figure 3: CCS histology differs from FAP
(A) Histological analysis of colonic biopsies from a familial polyposis (FAP) patient compared to a non-disease control. Images were taken at 40X (Scale = 100µm). (B) Measurements were taken from 50 crypts using the Nikon Imaging Software tool to measure the diameter. Methodology is depicted in Supplemental Figure 1C. (C) Crypt diameter was measured comparing images from the Non-CCS, to CCS 1 and 2, and FAP colonic biopsies. (D) Immunostaining for enteroendocrine (CHGA) cells was performed on paraffin embedded colonic sections from CCS1 and FAP tissues (GREEN). Images were taken at 40X (Scale = 100µm). ****, $p<0.0001$ determined using an unpaired two-tailed t-test.

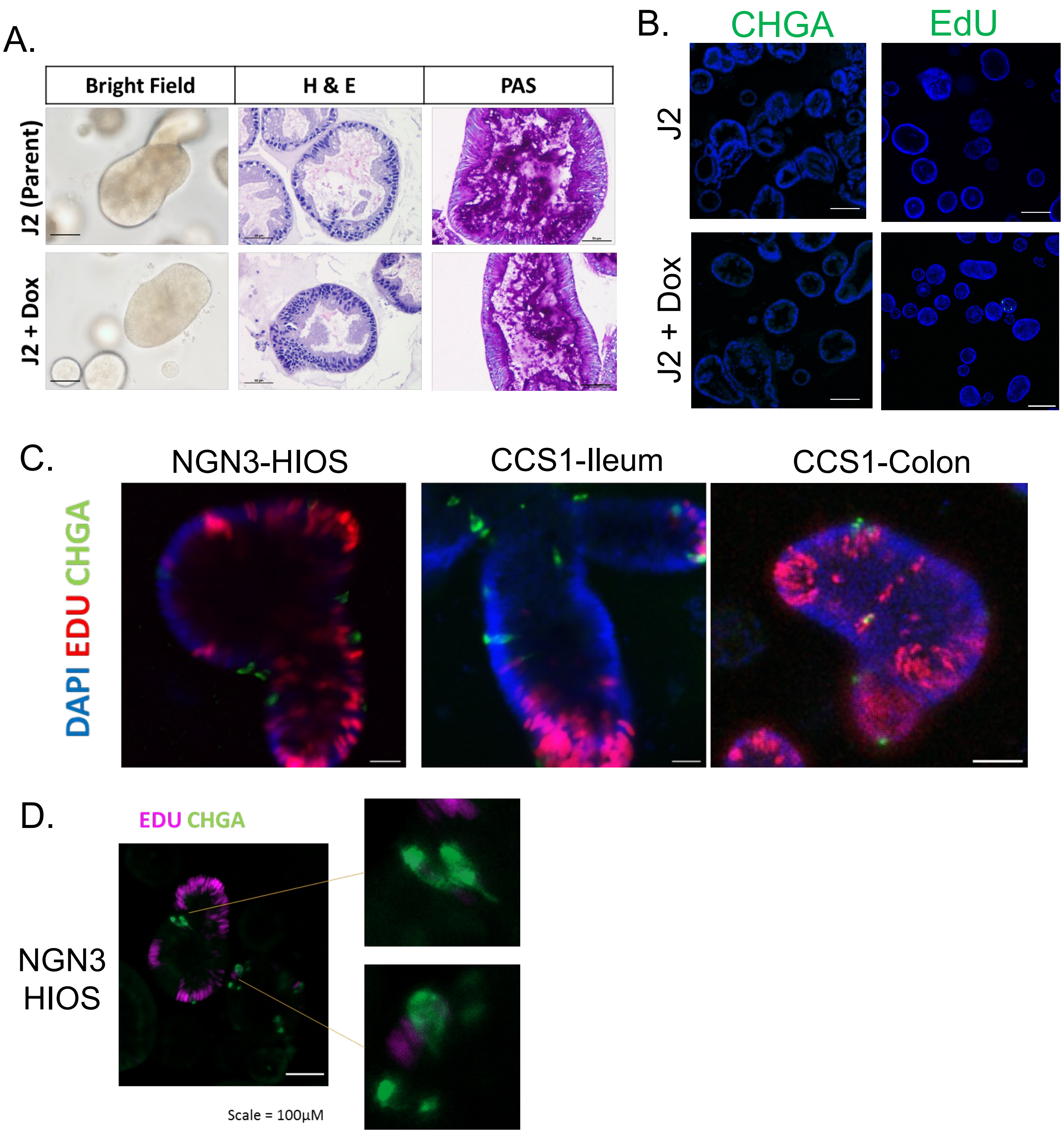

Supplementary Figure 4: Doxycycline does not induce proliferation in wildtype HIOs and EECs themselves are not proliferative

HIOs were treated with or without doxycycline for 5 days. Doxycycline alone had no affect on HIO morphology or proliferation in the wildtype HIO s. (A) Bright field, H&E, and PAS staining did not indicate unique morphology or increased secretory cells. Images were taken at 20X (Scale = 150µm). (B) Immunostaining for chromogranin A (CHGA), green cells, showed no CHGA+ cells. Cell nuclei were stained with DAPI (blue). To assess proliferation, EdU was added 24 hours prior to immunostaining. EdU uptake was detected using ClickIT® technology. Images were taken at 20X (Scale = 150µm). (C) NGN3 and CCS1 HIOs were co-stained with CHGA (GREEN) and EdU (RED) to determine if CHGA+ cells were proliferating. Cell nuclei were stained with DAPI (BLUE). CHGA+ cells are not proliferating. Images were taken at 40X (Scale = 100µm). (D) High power magnification of CHGA+ and EDU+ cells. Images are shown in 1 planar view of a 3D organoid.

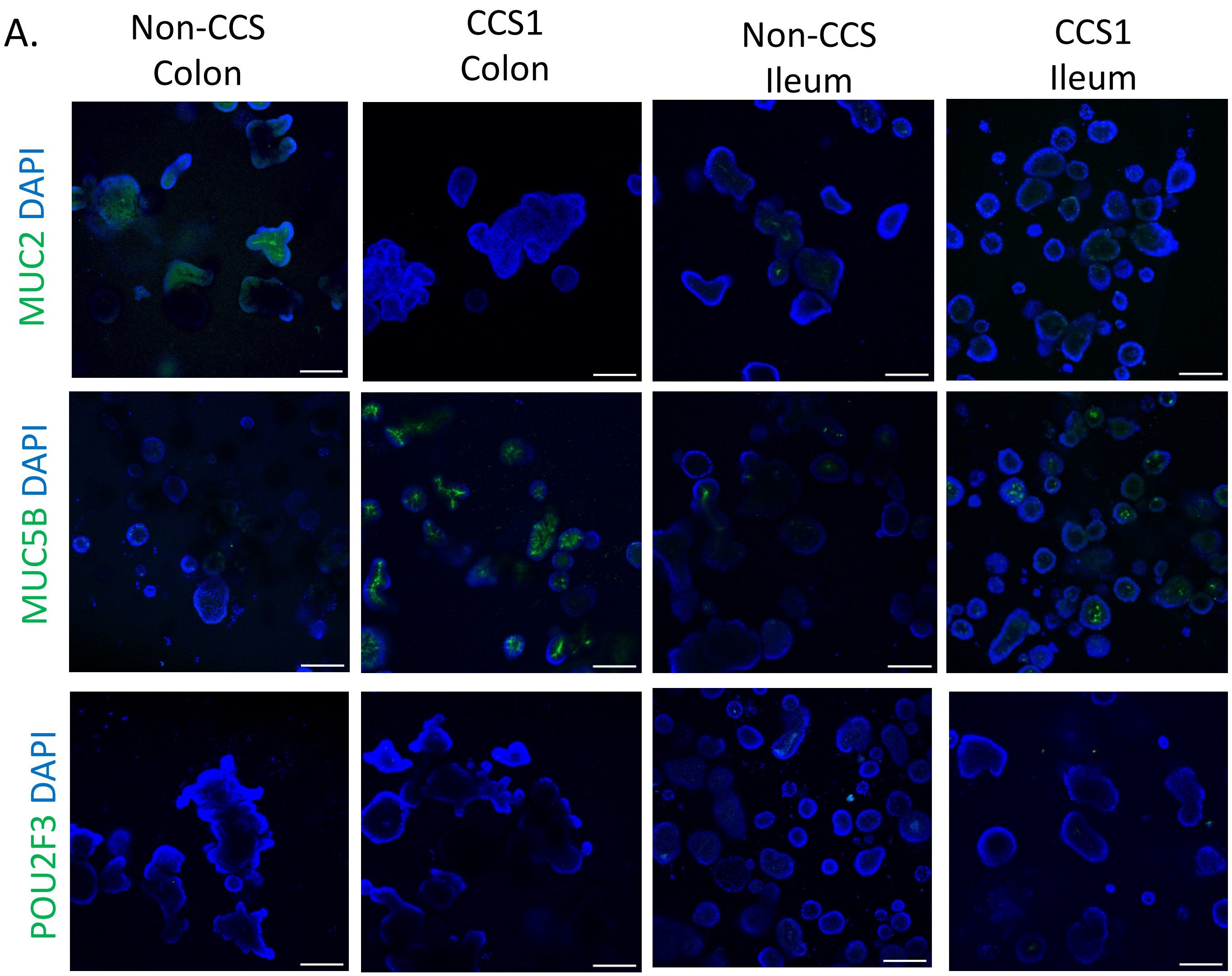

Supplementary Figure 5: Additional Secretory Cell Analysis of CCS and NGN3 HIOs

(A) HIOs were assessed for MUC2 using a MUC2 antibody (Santa Cruz sc-515032), MUC5B using a MUC5B antibody (Millipore HPA008246), and POU2F3 with a POU2F3 antibody (Sigma HPA019652). Images were taken at 10X (Scale = 200μm).

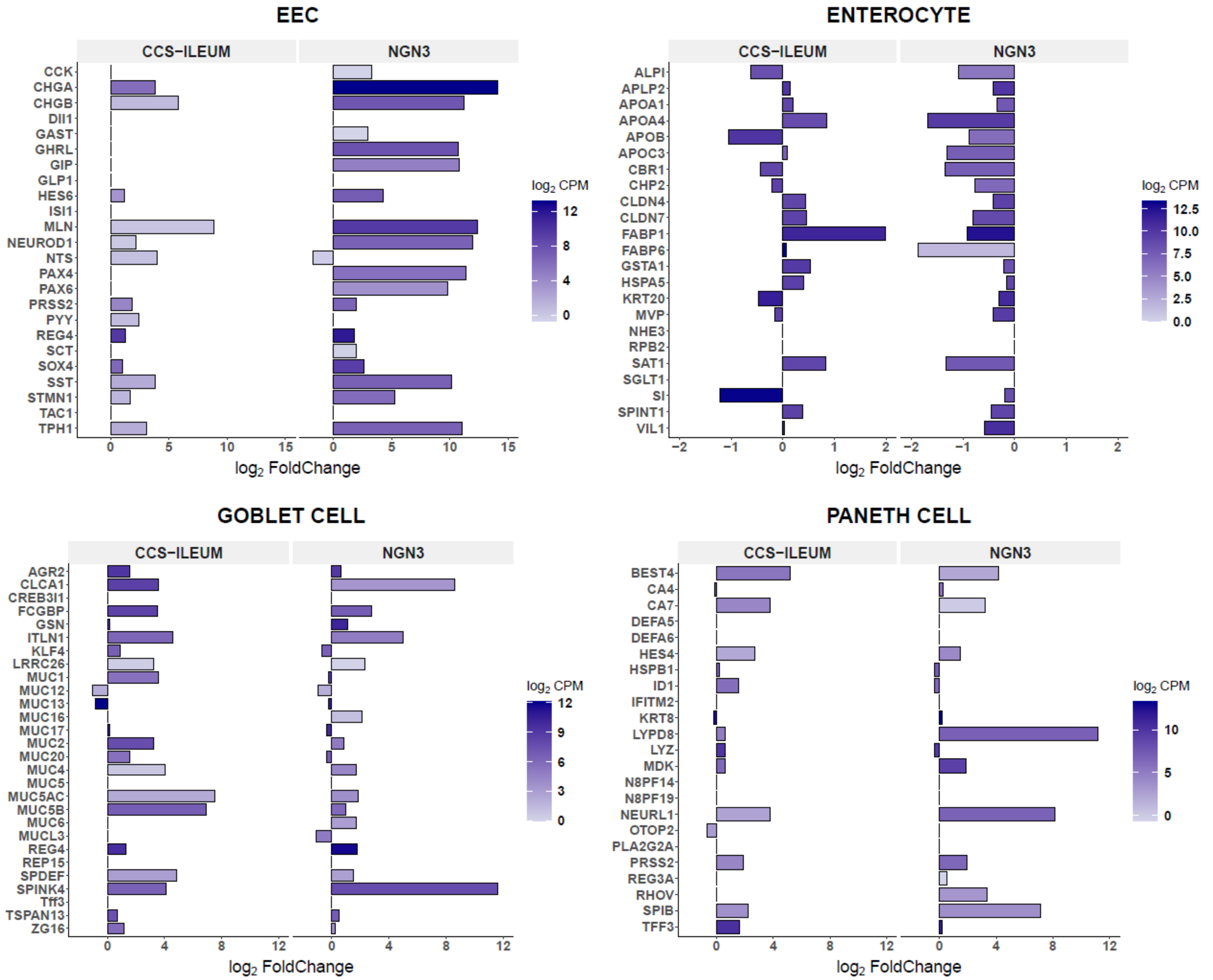

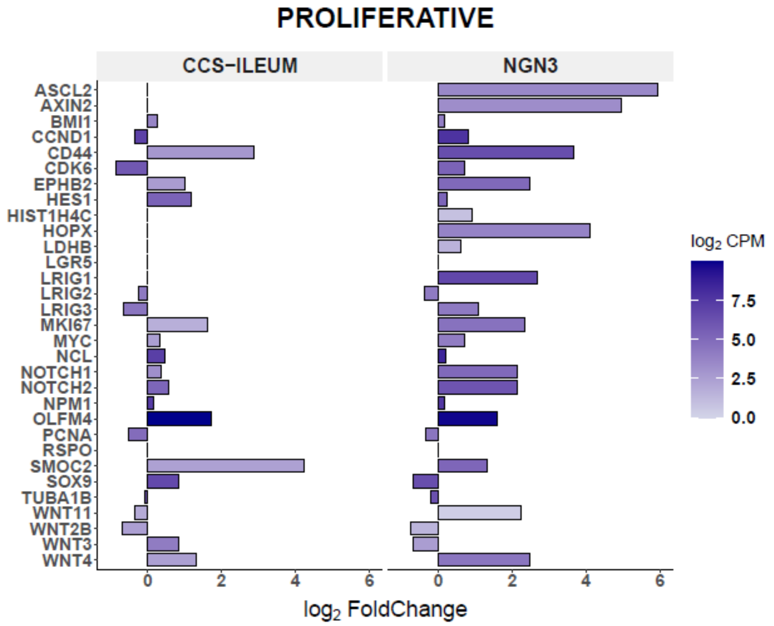

Supplementary Figure 6: Transcriptional analysis of epithelial cell type specific markers Known markers for each of the small intestinal epithelial cell types was assessed transcriptionally for CCS1-Ileum and NGN3 HIOs. Transcript levels for each gene are shown as the $\log_2$CPM compared to their respective controls.

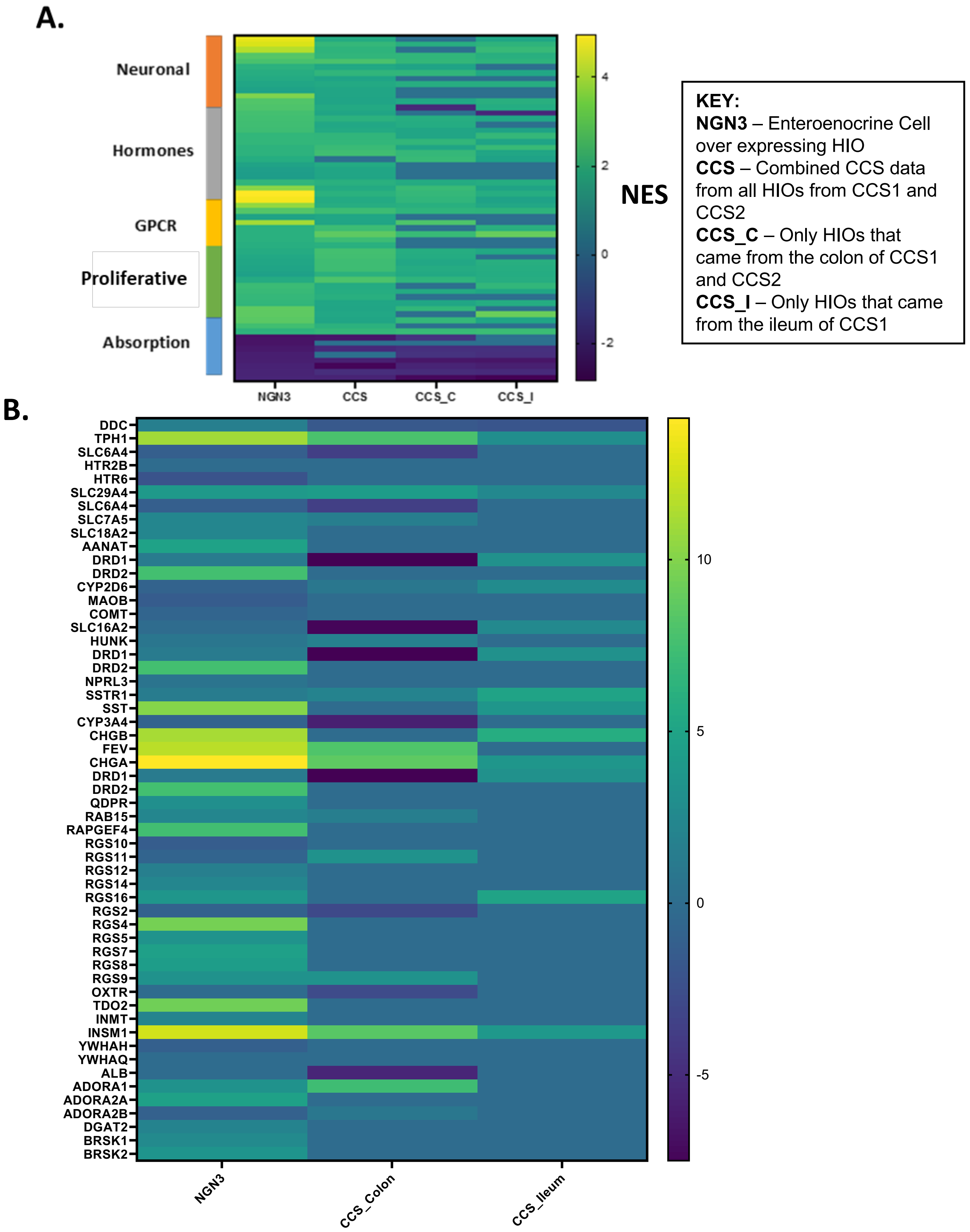

Supplemental Figure 7: GSEA Pathway Analysis and 5HT related genes in CCS and NGN3 HIOs (A) The up-regulated (FDR ≤ 0.25) Reactome pathways common between the CCS and NGN3 HIOs with normalized enrichment score (NES) based on the changes in gene expression. (B) List of genes involved in the synthesis or signaling of serotonin plotted as $\log_2$ fold change in CCS and NGN3 HIOs.

A.

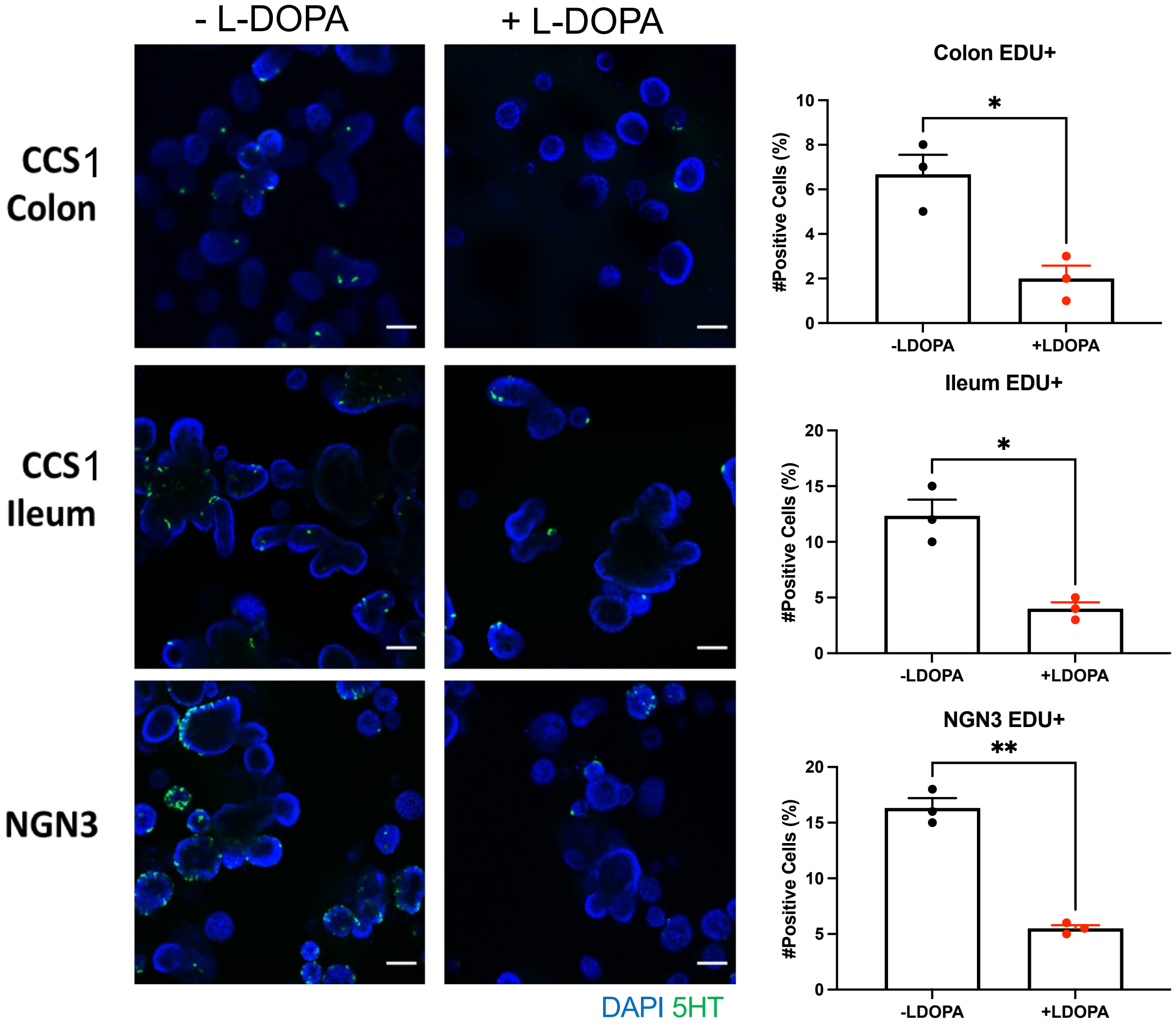

B.

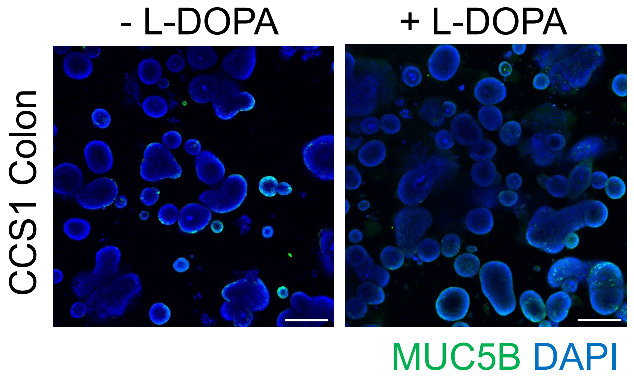

Supplemental Figure 8: Effects of serotonin (5HT) Inhibition.
(A) CCS HIOs were treated with 5HT inhibitor, L-DOPA, for 48 hours. Following L-DOPA treatment, HIOs were stained with an anti-5HT antibody to confirm L-DOPA decreased 5HT. 5HT (GREEN) DAPI (BLUE). 5HT positive cells were quantified by flow cytometry before and after L-DOPA treatment confirming the decrease in 5HT positive cells. Images were taken at 20X (Scale = 150μm). (B) Treatment with L-DOPA did not affect MUC5B production in the CCS Colon HIOs. Bars represent mean + SD (n=3). Images were taken at 10X (Scale = 200μm). **, $p<0.005$, *, $p<0.05$ determined using an unpaired two-tailed t-test with Welch's correction.

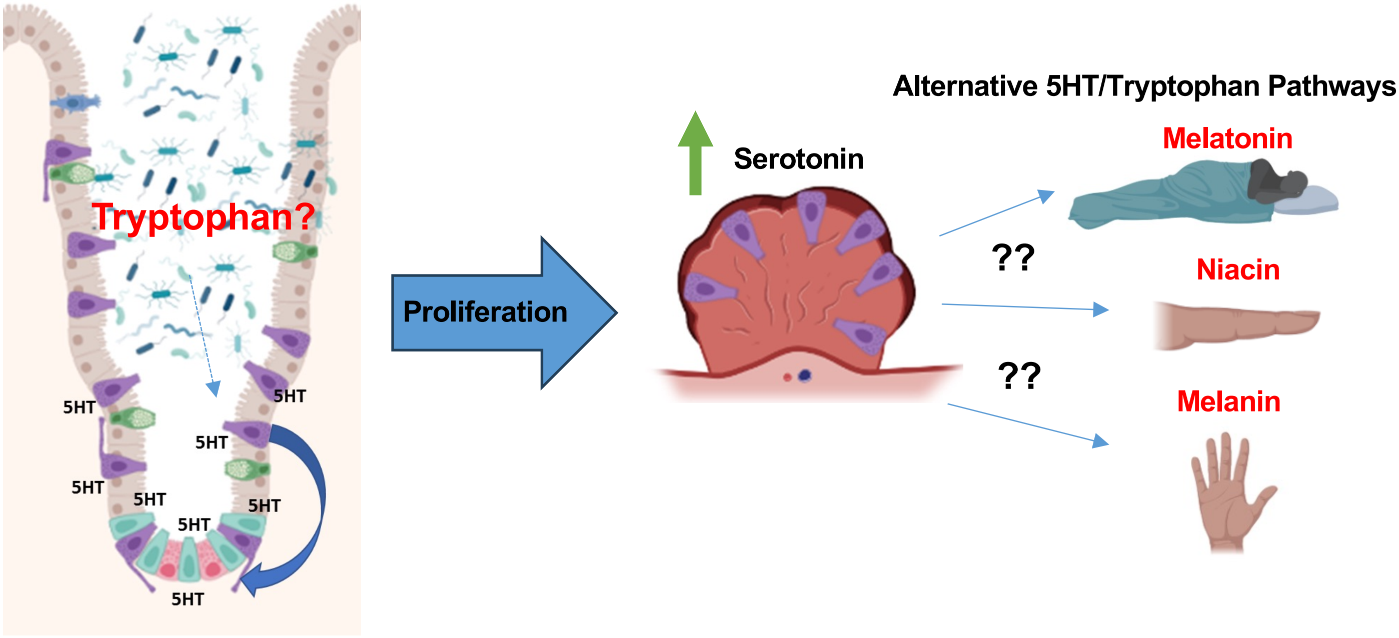

Supplemental Figure 9: Working model of CCS pathogenesis

Alternations in the CCS microbiome may be contributing the excess of tryptophan that is needed by eneteroendocrine cells (also in excess) to make serotonin. In turn the excess serotonin results in increased levels of polyposis and proliferation. Melatonin is a down stream product of serotonin and may contribute to the patients feeling of tiredness. Further, an alternative pathway for tryptophan instead of serotonin production is niacin which is essential for nail formation which the patients also lack. It is possible that the shift of increased serotonin production takes away from niacin synthesis. Melanin is also indirectly linked to these pathways which may explain the skin discoloration seen in CCS patients. Created with Biorender.com.

A.

	Averaged Taxa	Shannon Index	Simpson Index
**CCS**	47.67	2.39	0.78
**FAP**	58.84	2.57	0.82
**Healthy**	66.47	2.71	0.86

B.

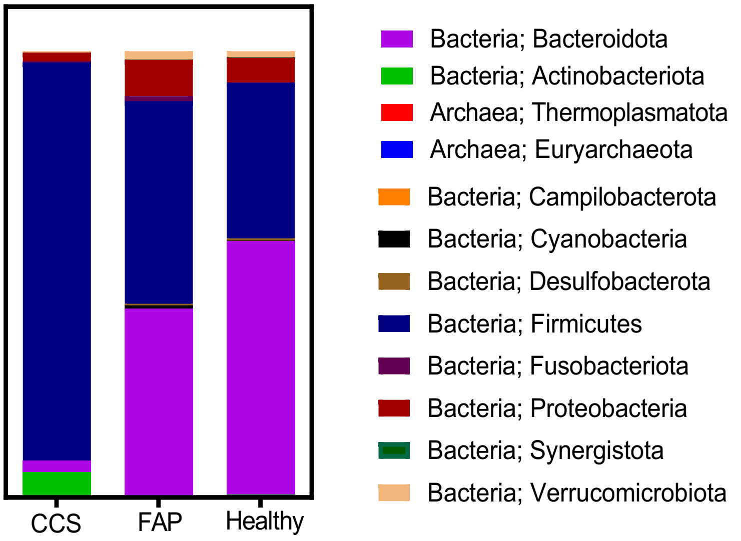

C.

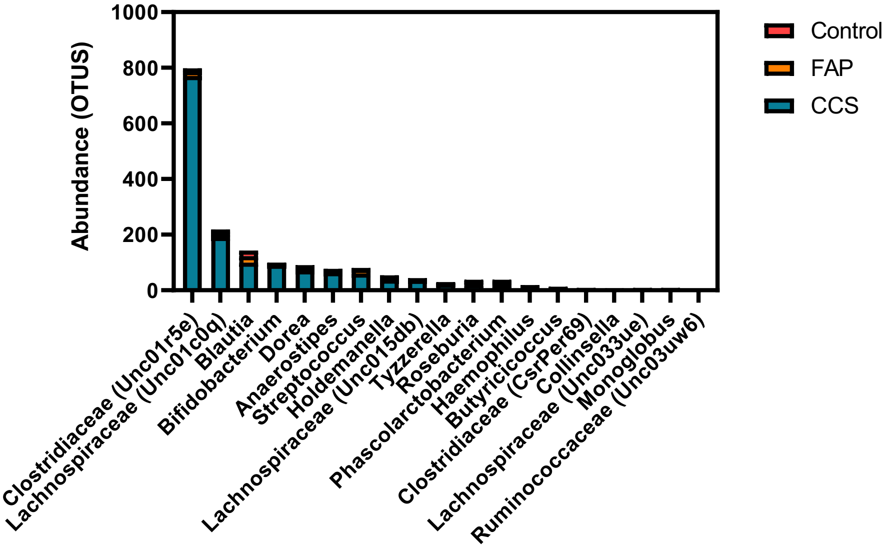

D.

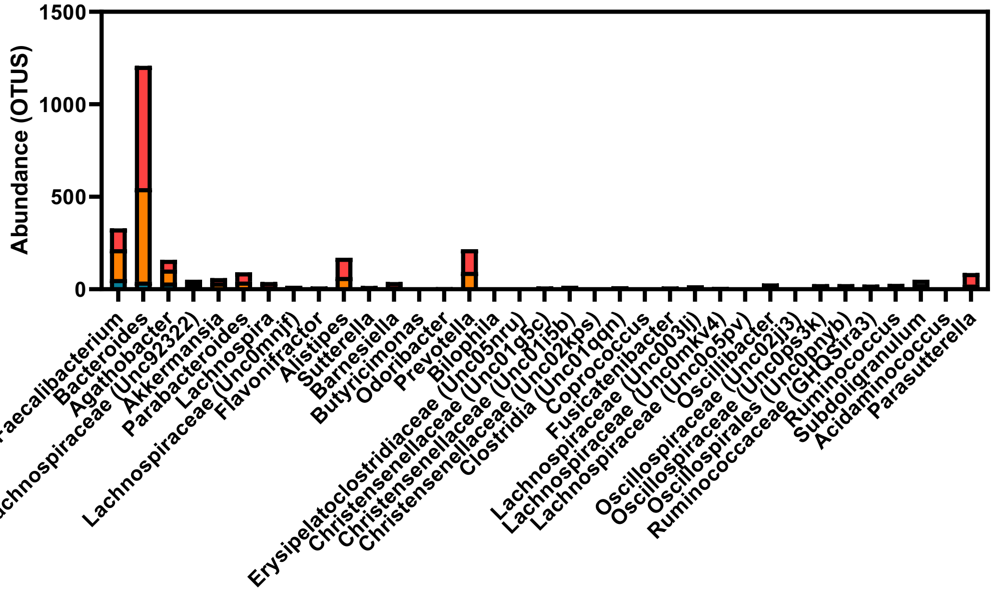

Supplemental Figure 10: CCS patients have alternations in their microbiome
3 technical replicates of CCS patient stool was submitted for 16S Microbial Profiling (n=3 samples), 19 samples from FAP patients, 19 healthy control samples. (A) Average diversity scores for different sets of samples. (B) Phylum level OTUs for the samples. (C) Bacteria genera upregulated in CCS compared to FAP and controls. (D) Genres downregulated in CCS compared to FAP and controls.
